# A review of multimodal deep learning methods for genomic-enabled prediction in plant breeding

**DOI:** 10.1093/genetics/iyae161

**Published:** 2024-11-05

**Authors:** Osval A Montesinos-López, Moises Chavira-Flores, Kismiantini, Leo Crespo-Herrera, Carolina Saint Piere, HuiHui Li, Roberto Fritsche-Neto, Khalid Al-Nowibet, Abelardo Montesinos-López, José Crossa

**Affiliations:** Facultad de Telemática, Universidad de Colima, Colima, Colima 28040, México; Instituto de Investigaciones en Matemáticas Aplicadas y Sistemas (IIMAS), Universidad Nacional Autónoma de México (UNAM), Ciudad de México 04510, México; Statistics Study Program, Universitas Negeri Yogyakarta, Yogyakarta, 55281 Yogyakarta, Indonesia; International Maize and Wheat Improvement Center (CIMMYT), Km 45, Carretera Mexico-Veracruz, Texcoco, CP 52640 Edo. de México, México; International Maize and Wheat Improvement Center (CIMMYT), Km 45, Carretera Mexico-Veracruz, Texcoco, CP 52640 Edo. de México, México; Institute of Crop Science Chinese Academy of Agricultural Sciences (CAAS), Chin Office, 12 Zhongguancun, South Street, Beijing 100081, China; Louisiana State University, Baton Rouge, LA 70803, USA; Department of Statistics and Operations Research, King Saud University, Riyah 11451, Saudi Arabia; Departamento de Matematicas, Centro Universitario de Ciencias Exactas e Ingenierías (CUCEI), Universidad de Guadalajara, 44430, Guadalajara, Jalisco, México; International Maize and Wheat Improvement Center (CIMMYT), Km 45, Carretera Mexico-Veracruz, Texcoco, CP 52640 Edo. de México, México; Louisiana State University, Baton Rouge, LA 70803, USA; Colegio de Postgraduados, Montecillos, Edo. de México CP 56230, México; Distinguish Scientist Fellowship Program, King Saud University, Riyah 11451, Saudi Arabia

**Keywords:** data fusion, genomic prediction, multimodal deep learning, plant breeding

## Abstract

Deep learning methods have been applied when working to enhance the prediction accuracy of traditional statistical methods in the field of plant breeding. Although deep learning seems to be a promising approach for genomic prediction, it has proven to have some limitations, since its conventional methods fail to leverage all available information. Multimodal deep learning methods aim to improve the predictive power of their unimodal counterparts by introducing several modalities (sources) of input information. In this review, we introduce some theoretical basic concepts of multimodal deep learning and provide a list of the most widely used neural network architectures in deep learning, as well as the available strategies to fuse data from different modalities. We mention some of the available computational resources for the practical implementation of multimodal deep learning problems. We finally performed a review of applications of multimodal deep learning to genomic selection in plant breeding and other related fields. We present a meta-picture of the practical performance of multimodal deep learning methods to highlight how these tools can help address complex problems in the field of plant breeding. We discussed some relevant considerations that researchers should keep in mind when applying multimodal deep learning methods. Multimodal deep learning holds significant potential for various fields, including genomic selection. While multimodal deep learning displays enhanced prediction capabilities over unimodal deep learning and other machine learning methods, it demands more computational resources. Multimodal deep learning effectively captures intermodal interactions, especially when integrating data from different sources. To apply multimodal deep learning in genomic selection, suitable architectures and fusion strategies must be chosen. It is relevant to keep in mind that multimodal deep learning, like unimodal deep learning, is a powerful tool but should be carefully applied. Given its predictive edge over traditional methods, multimodal deep learning is valuable in addressing challenges in plant breeding and food security amid a growing global population.

## Introduction

The development of plant breeding (PB) strategies has significantly contributed to meeting the needs of the human population, which reached 3 billion by 1960, prior to the well-known Green Revolution (He and Li, 2020). Today, we face a similarly critical situation where advances in this field are poised to play a leading role, given the current climate crisis and the increasing demand for land, water, and energy to produce high-quality food for a global population projected to reach 9 billion by 2,050 (Ehrlich and Harte 2015). To meet this rising demand without expanding land use and to mitigate the adverse environmental impacts of climate change, it is imperative to adopt quicker and more efficient breeding strategies. Like many other research areas, PB has benefited from technological advances, particularly from machine learning (ML) and other branches of Artificial Intelligence (AI) (van Dijk *et al*. 2020). Not long ago, the application of deep learning (DL) methods to PB was considered an unexplored field (Ma *et al*. 2018). However, today, ML techniques, including DL methods, are extensively applied in PB studies. These methods are used to analyze and evaluate the transmission of information from DNA sequences to observable phenotypic traits in plants (Niazian and Niedbała 2020).

Several empirical studies have demonstrated the power of DL methods in applications to genomic selection (GS). The following examples illustrate how DL can enhance the predictive capabilities of conventional statistical methods. Ma *et al*. (2018) employed a convolutional neural networks (CNNs) to predict phenotypes from genotypic information, showing that this approach can be effectively combined with ridge regression—best linear unbiased predictor (RR-BLUP) methods. Montesinos-López, Montesinos-López, Crossa, *et al*. (2018), Montesinos-López, Montesinos-López, Gianola, *et al*. (2018) compared Genomic Best Linear Unbiased Predictor (GBLUP) and DL methods across 9 datasets, finding that DL outperformed GBLUP in 6 cases in terms of prediction accuracy. Additionally, Montesinos-López, Montesinos-López, Crossa, *et al*. (2018), Montesinos-López, Montesinos-López, Gianola, *et al*. (2018) compared 2 multitrait methods: a multitrait deep learning (MTDL) model and the Bayesian multitrait, and multienvironment model proposed by Montesinos-López *et al*. (2016), which is a multitrait version of GBLUP. Their findings suggest that the MTDL model is highly competitive for GS prediction.

Pérez-Rodríguez *et al*. (2020) introduced a Bayesian neural network (NN) designed to model ordinal data using a data augmentation approach. Evaluating the model's predictive ability with performance measures such as the Brier Score, Misclassification Error Rate, mean absolute error (MAE), and Spearman’s correlation coefficient, the authors found that their NN outperformed the widely used Bayesian ordered probit linear model for ordinal data analysis. Sandhu *et al*. (2021) compared a CNN, a multilayer perceptron (MLP), and an RR-BLUP method using data from a spring wheat nested association mapping population. They found that both DL methods outperformed RR-BLUP, achieving a 0 to 5% better prediction accuracy. Jubair *et al*. (2021) compared the performance of a DL method called GPTransformer, some statistical methods, and a Residual Fully Connected NN in predicting resistance to *Fusarium graminearum* from barley genotypic data. Their results indicated the potential of GPTransformer as an alternative to the popular BLUP (or RR-BLUP) model for genomic prediction of *Fusarium* head blight disease and mycotoxin presence.

Wang *et al*. (2023) used neural network genomic prediction (DNNGP) to integrate multi-omics data in plants. Analyzing datasets for wheat, maize, and tomato, they compared DNNGP with GBLUP, LightGBM, support vector regression (SVR), Deep Learning Genomic Selection (DeepGS), and Deep Learning Genome-Wide Association Study (DLGWAS) across various sample sizes. Overall, DNNGP performed equally well or better than commonly used linear models (GBLUP), ML-based methods (LightGBM and SVR), and DL-based methods (DLGWAS and DeepGS) across a wide range of prediction tasks. Additionally, its runtime was comparable to GBLUP, LightGBM, SVR, and DLGWAS, and ∼10 times faster than DeepGS.

Applications of DL methods in GS have gained popularity in recent years, and it is reasonable to expect this trend to continue. However, like other techniques, conventional DL methods have some limitations and do not always outperform classical statistical methods such as the popular GBLUP and other ML approaches. For instance, Bellot *et al*. (2018) assessed the ability of CNN and MLP in predicting complex human traits and found that, in general, CNN performance was competitive with linear models like BayesB (Meuwissen *et al*. 2001) and Bayesian Ridge Regression (BRR) (Henderson 1984), although DL did not consistently outperform these models by a significant margin.

A study by Zingaretti *et al*. (2020) analyzing blueberry and strawberry experimental data found that DL methods did not offer advantages over linear models, except when the epistasis component played a significant role. In cases where nonlinear effects were significant, Bayesian linear models [Bayesian Lasso (BL) and BRR] could achieve or even surpass the predictive accuracy of DL methods. Additionally, Abdollahi-Arpanahi *et al*. (2020) reported that DL methods did not improve prediction accuracy compared to Gradient Boosting, random forest (RF), GBLUP, and Bayes B.

Montesinos-López *et al*. (2021) provided a comprehensive review and comparison of these and other studies, concluding that although DL algorithms are more efficient at capturing nonlinear patterns than conventional genomic prediction methods, they do not demonstrate clear superiority in prediction power compared to traditional genome-based prediction models.

All studies mentioned so far focus solely on using genotypic information to predict traits of interest in different species. This approach has the obvious disadvantage of overlooking additional information, such as environmental or phenotypic data, which could enhance prediction accuracy. Multimodal deep learning (MMDL) is a MLML approach that aims to achieve similar goals as unimodal conventional DL methods but stands out by integrating diverse sources of information into the modeling process. MMDL has been applied in various research and industry areas, including face authentication (Ding and Tao 2015), biometric recognition (Alay and Al-Baity 2020; Aung *et al*. 2022), emotion recognition (Liu *et al*. 2021), and autonomous driving systems (Aranjuelo *et al*. 2018; Ennajar *et al*. 2021; Elallid *et al*. 2022).

MMDL often uses data fusion to combine information from multiple sources or modalities. Data fusion in this context refers to integrating different types of data, such as images, text, audio, and other sensory inputs, to improve the performance and robustness of a ML model. Using data fusion in MMDL helps to leverage complementary information from multiple sources, leading to more accurate, robust, and comprehensive models. This approach is particularly beneficial in tasks such as image captioning, audio–visual speech recognition, and multimodal sentiment analysis, where integrating different types of data can provide a more complete understanding of the input.

Fusion of genomic data with other modalities offers significant potential for advancing PB. Here are specific examples related to fusion strategies involving genomic data in the context of PB: (1) Environmental information: Integrating genomic data with environmental data can enhance the accuracy of the GS methodology. (2) Transcriptomic Data Fusion: Combining genomic variants with gene expression profiles to improve the prediction of the models. (3) Epigenomic data fusion: Integrating genomic variants with epigenomic marks to study gene regulation and disease mechanisms. (4) Multi-omics data fusion: Integrating genomic data with other omics data to comprehensively characterize biological systems. By integrating genomic data with other modalities and considering these adaptations, researchers can gain deeper insights into biological mechanisms and improve the prediction of complex traits.

In this study, we provide a comprehensive review of MMDL applications for genomic prediction, highlighting their potential in terms of prediction performance compared to traditional linear and nonlinear ML methods. We begin with an introduction to fundamental concepts of neural networks and present a list of popular NN architectures. We then explore strategies for combining different types of data in DL models. Additionally, we address considerations for tuning hyperparameters, mentioning some frameworks available for this task, as well as frameworks for the computational implementation of MMDL models. Finally, we discuss key aspects to keep in mind when applying MMDL methods.

## Why ML for genomic prediction?

Genomic prediction, in the context used here, refers to developing models that predict breeding values from genotypic data, typically derived from single nucleotide polymorphism (SNP) data. Breeding values represent the genetic merit of individuals based on their genotype, which is critical for selecting superior individuals in plant and animal breeding programs. Traditional methods like RR-BLUP, have been widely used for genomic prediction. These linear models are effective in capturing additive genetic effects and are computationally efficient. As the complexity of genotypic, phenotypic, and environmental data increases, there is a growing need for investigating more flexible approaches that can capture complex interactions and patterns.

ML models, including DL, offer several advantages over traditional statistical methods for genomic prediction. They can capture complex, nonlinear relationships between genotypic data and phenotypic outcomes, which linear models might miss. Additionally, ML models can integrate data from multiple sources (e.g. genomic, phenotypic, and environmental), providing a more comprehensive view of the factors influencing breeding values. As a result, ML approaches have the potential to improve the accuracy of predictions, especially in scenarios where traditional models face limitations.

The RR-BLUP model as presented by Ma *et al*. (2018) is


y=1b+Xg+e,

where e is a vector of error terms, y is the *n*-dimensional vector of phenotypic values, 1 a *n*-dimensional vector of ones, ***b*** is the overall mean, X the n×p matrix of genotype scores coded as the number of copies of the minor allele in a specific position, g is a *p*-dimensional vector of marker effects with assumed zero mean normal distribution and genetic variance–covariance matrix $\sigma_{g}^{\;2}I$ where I is an p×p matrix and $\sigma_{g}^{\;2}$ is the marker effect variance. The most obvious limitation of this model is that it is only able to capture linear relationships between genotypes and phenotypic traits and because GS is a predictive methodology, other ML models have been applied to handle this limitation, such as random forests (Chen and Ishwaran 2012), support vector machines (Zhao *et al*. 2020), or gradient boosting machines (Zhou *et al*. 2023). However, as pointed out by Mahood *et al*. (2020), all of these mentioned methods require explicit specification of various input features, whereas NN models are able to extract features from the training data automatically. But on the other hand, while those traditional ML models work acceptably with a hundred input training data samples, NN models require thousands to millions of input data samples for accurate working. Nonetheless, despite this apparent limitation, the success of NN models lies in their observed capacity to learn well from large datasets even if these have a high number of features (Goodfellow *et al*. 2016).

### What is MMDL?

To understand what MMDL is, consider the following physical analogy provided by Akkus *et al*. (2023). Humans have 5 primary senses: hearing, touch, smell, taste, and sight. Through these modalities, human beings perceive the world around them. Thus, “multimodal”? refers to the simultaneous integration of diverse channels of information to gain a comprehensive understanding of the environment. In other words, “multimodal” refers to the inclusion (fusing) of data of various types, such as audio, images, and text, in a single DL NN model. Current computational technologies enable the implementation of such approaches, even for the large volumes of data characteristic of genomic information. In the context of GS with multimodal DL, we can integrate genomic, phenomic, environmental, proteomic, and other omics data within each modality.

## Architectures of DL models

It is important to note that MMDL techniques utilize many of the same tools as their unimodal counterparts. Therefore, it is helpful to revisit some of the most popular DL architectures. Although we assume the reader is somewhat familiar with DL algorithms, we provide in Supplementary material an extensive review of the most relevant NN architectures, offering intuitive explanations of how MLP, CNNs, recurrent neural networks (RNNs), and others work. For readers interested in a deeper understanding of neural networks, we recommend comprehensive texts such as Goodfellow *et al*. (2016), Aggarwal (2018), Zhang, Lipton, *et al*. (2021), and Zhang, Zhang, *et al*. (2021). Additionally, we highlight examples where these architectures have been applied in the context of genomic-enabled prediction.

## Artificial neurons

**Fig. 1.**
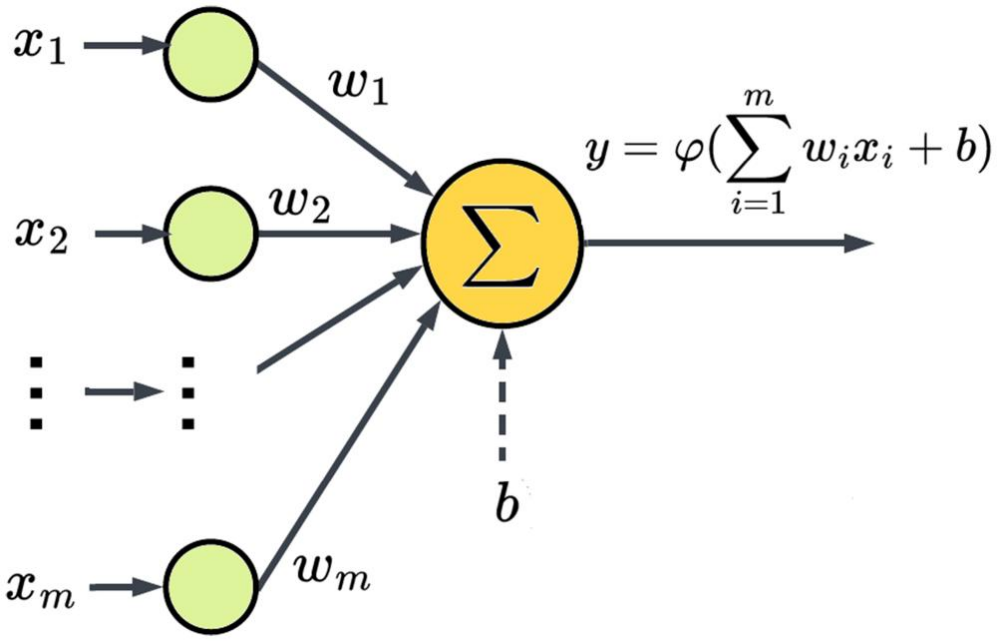
Basic anatomy and working of an artificial neuron.

**Fig. 2.**
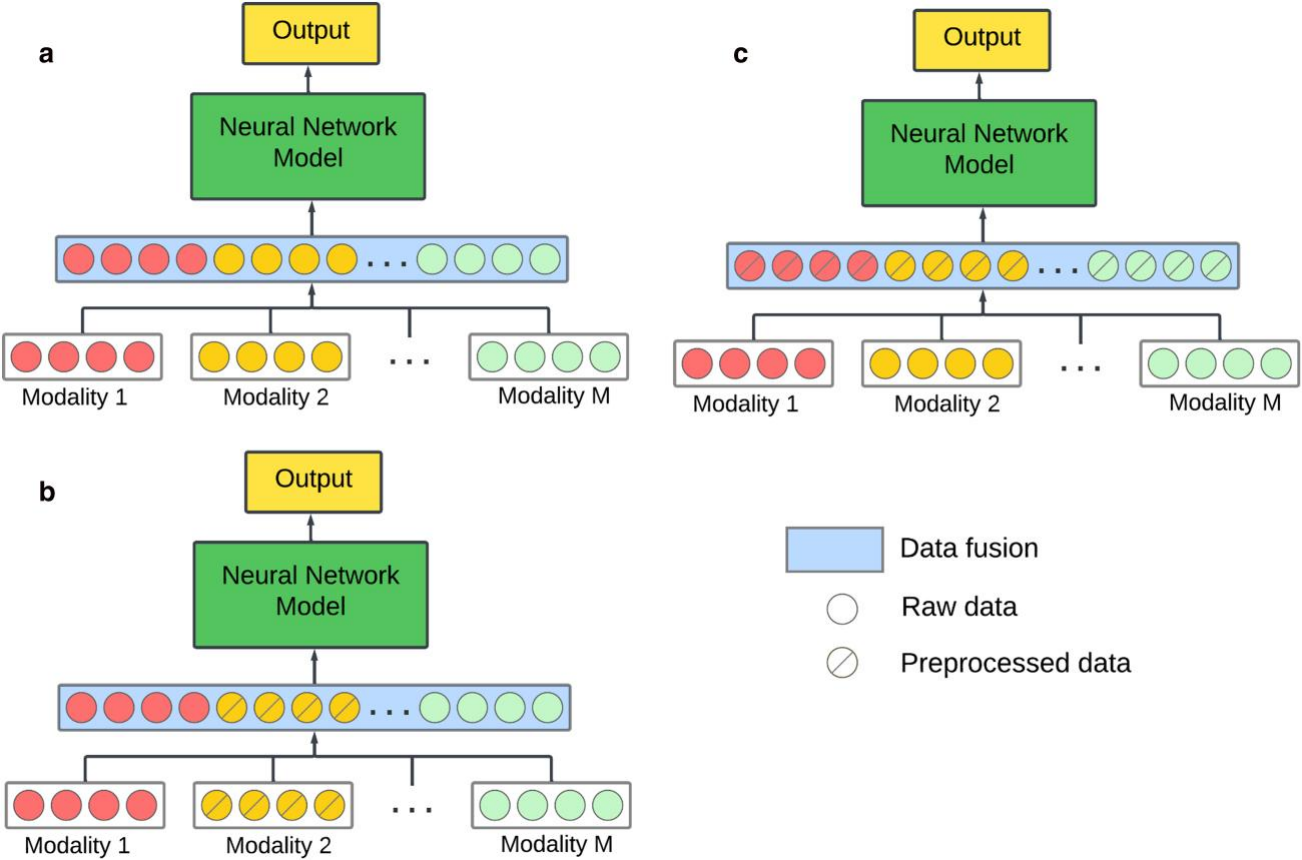
a) Early data fusion with raw data for all modalities; b) early data fusion with some modalities preprocessed separately; and c) early data fusion with all modalities preprocessed jointly.

**Fig. 3.**
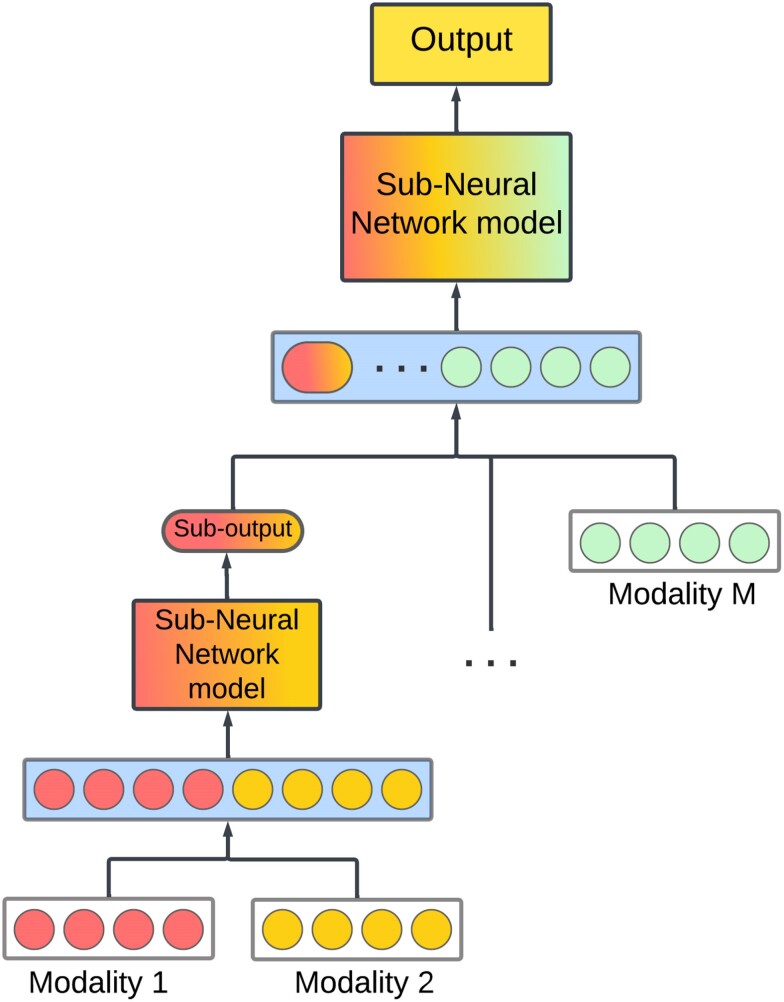
Example of a model with intermediate data fusion. Note that after the fusion module where representations for all modalities are combined, we have a final sub-model to produce the definite output. The Sub-output means a *marginal representation* (marginal latent factor), that is, representations of data features specific to each modality.

**Fig. 4.**
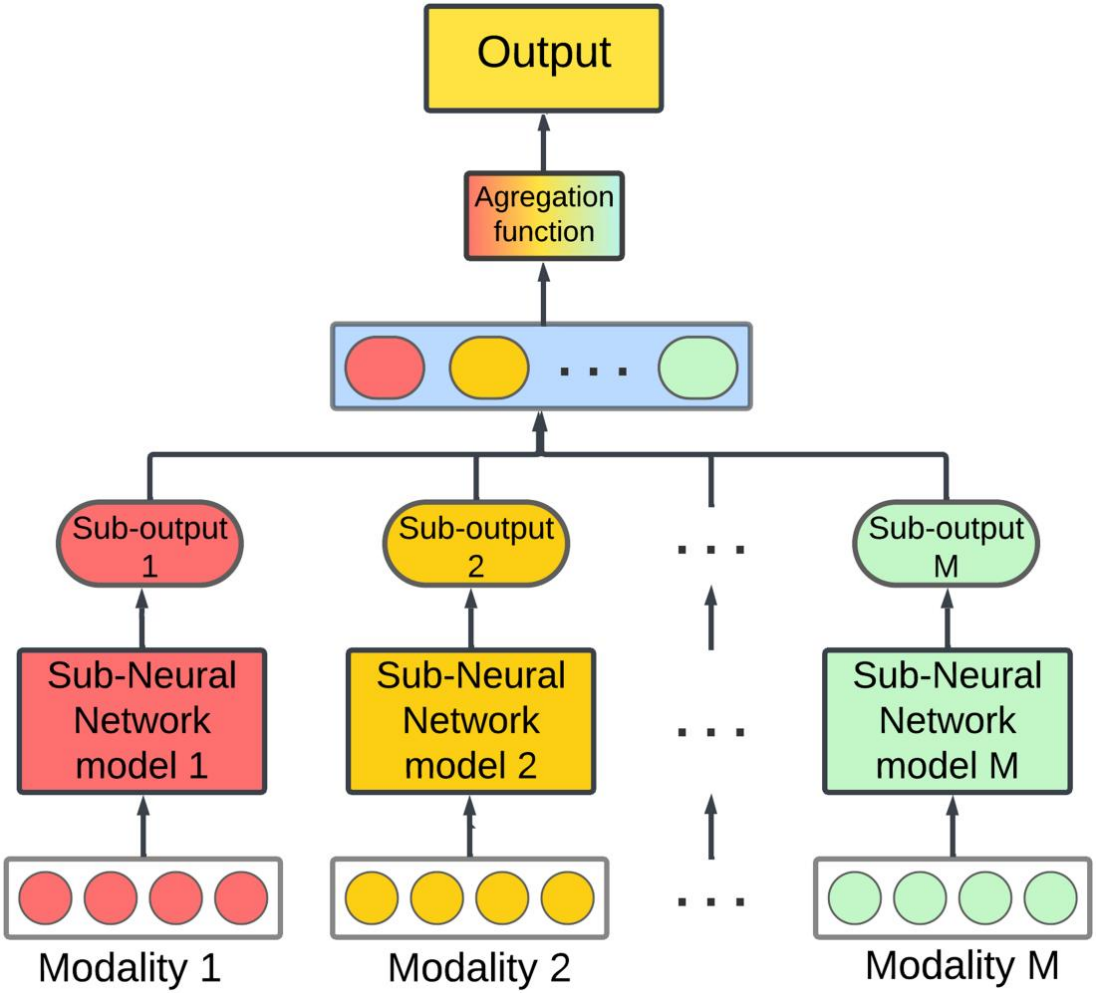
General form of late data fusion in MMDL. Every modality has its own separated DL model. The resulting outputs are concatenated and finally combined by an aggregation function to get the final output.

The *artificial neuron* (AN) is the basic *unit* of a NN model, and it aims to emulate the working of the human brain, where every neuron is part of a nervous network and reacts to stimuli coming from other neurons or from external sources producing a stimulus which is transmitted to other neurons in the network by connections known as “synapses”? (Zayegh, 2018). The mathematical formulation of an AN (Fig. 1) is as follows:


(1) $$y=\varphi \left(\sum_{i=1}^{m}w_{i}x_{i}+b\right)=\varphi (w^{T}x+b)$$

where *y* is a target *output* to be predicted, for example, an observable phenotypic trait in wheat or maize, $x=(x_{1},\ldots ,x_{m}{)}^{T}$ are the *input signals* or stimuli, for example, genotypes as SNPs data, $w=(w_{1},\ldots ,w_{m}{)}^{T}$ are the *synaptic weights*, *b* is a *bias* term, and φ is an *activation function*. Note that when φ is the identity function, we have the form of the expectation of a linear regression model. Details of commonly used DL architectures are given in Supplementary Tables 1–6 and Figs. 1–9).

## Activation functions

The purpose of activation functions is to capture nonlinear patterns in an NN model. The most popular activation functions are linear, rectified Linear Unit (ReLU), Leaky ReLU, threshold, sigmoid, hyperbolic tangent, exponential, and softmax. In this, we include the mathematical formulation of all the activation functions mentioned (Table 1).

**Table 1.** Some of the popular activation functions and their mathematical formulations.

Function	Formula
Linear	φ(x)=x
Rectified Linear Unit (ReLU)	φ(x)=max(0,x)
Leaky ReLU	φ(x)=x if x≥0, and φ(x)=αx if x<0, where 0<α<1
Threshold	φ(x)=1 if x>0, φ(x)=0 otherwise
Sigmoid	$\varphi (x)=\frac{1}{1+\exp(-x)}$
Hyperbolic tangent	$\varphi (x)=\tan h(x)=\frac{\exp(x)-\exp(-x)}{\exp(x)+\exp(-x)}$
Exponential	φ(x)=exp(x)
Softmax	$\varphi (z_{i})=\frac{\exp(z_{i})}{\sum_{\;j=1}^{C}\exp(z_{j})},$ where $z_{1},\ldots ,z_{C}$ are the outputs from the previous layer

This does not intend to be an exhaustive list of activation functions.

Montesinos-López *et al*. (2021) present a brief review of activation functions and some suggestions about when to use each one of them for specific tasks. The reader interested in this subject in greater detail can find more by Aggarwal (2018), Zhang, Lipton, *et al*. (2021), Zhang, Zhang, *et al*. (2021), Akkus *et al*. (2023).

## Advanced DL models and their applications for genomic prediction in PB

Advanced models such as RNNs, Residual Network (ResNet), Transformers, Graph Neural Network (GNN), and Autoencoders are powerful tools for genomic prediction in PB. Important practical questions for plant breeders are (1) why these models are suitable for genomic prediction and (2) how to perform genomic prediction using these models. The overall implications of these advance models in practical PB include (1) significant improvement of the accuracy of genomic predictions by effectively modeling complex genetic architectures; (2) increase speed and efficiency by better feature extraction and representation learning capabilities, these models can provide faster and more efficient predictions, accelerating the breeding process; (3) each type of NN can be tailored to specific aspects of genomic data, allowing breeders to choose the most appropriate model for their specific prediction tasks. They provide advanced methods for handling high-dimensional data, capturing complex relationships, and improving prediction accuracy, ultimately aiding in the development of better crop varieties

Using these advanced genomic prediction methods allows plant breeders to make more informed decisions, speeding up the breeding process and enhancing the development of crop varieties with improved performance, resilience, and yield. Details of these architectures are provided in Supplementary material.

## Some considerations on selecting an appropriate DL architecture

Guidance on how to choose an ad hoc architecture is worth including. Important attention should be paid to the gradient vanishing (GV) problem, which can lead to a lack of trainability of a model. As mentioned, some architectures are designed to handle this problem, but whether GV appears or not also depends on the appropriate choice of activation functions. For example, it is well known that the output of the derivative of the sigmoid function tends to be zero for higher and lower inputs, which can lead to the GV problem (Dubey *et al*. (2021). The ReLU activation function can be used to tackle this situation, since its linear component for nonzero values can help to protect gradients from becoming too small. As mentioned, residual networks or long short term memory (LSTM) models are thought to solve this problem, but LSTM, as they are essentially recurrent networks, require many synaptic connections, which could increase the computational cost.

It should be noted that MLPs have the advantage of not assuming a particular structure in the input features (Goodfellow *et al*. 2016), which could be leveraged, for example, in a PB context, where some environmental factors like soil pH level, soil moisture, and temperature, could be regarded at first glance as independent, making appropriate the use of MLPs to process them jointly. On the other hand, RNNs and their variants, for example, LSTM, should be taken into account when dealing with sequential input data. In the case of CNNs, although their applications are mostly found in the field of computer vision, we have briefly mentioned how they can be used to process genomic data. Transformers can also be used to exploit the sequential structure of DNA, as demonstrated by Jubair *et al*. (2021) and Måløy *et al*. (2021). However, simply stacking more layers in vanilla transformers does not necessarily benefit the model and can lead to gradient degeneration, as pointed out by Wang *et al*. (2019).

## Strategies for the fusion of data from different modalities

A relevant challenge in multimodal tasks involves determining the most effective approach to combine data coming from different modalities (Akkus *et al*. 2023). MMDL models can be classified by the stage of the learning process in which they merge data coming from different information channels (modalities), such as early data fusion models, intermediate data fusion models, or late fusion models. A meticulous description of this topic can be found in the studies by Huang *et al* (2020), Boulahia *et al*. (2021), and Stahlschmidt *et al*. (2022), and to a lesser extent in Liu *et al*. (2018). We extract some relevant concepts about what each one of these three fusion strategies consists of from such works. This classification of data fusion strategies is indifferent to the type of NN used and to the nature of data involved in every situation. All these 3 approaches allow the integration and leverage of a diversity of learning mechanisms, depending on the particular purpose.

## Early data fusion

This strategy consists of merging data from different modalities in the first stage of the learning process. Data from different modalities can be concatenated in the same vector or matrix, which is used by the DL model for the input layer (see Fig. 2).

An advantage of this fusion approach is that it allows us to jointly leverage the input information from the different modalities with a fully connected layer at the beginning, especially when the ordering of the input features is irrelevant to the task in question. On the other hand, when the ordering of the input features carries structural information, such as in time series or genomic information, recurrent layers or convolutional layers can be used for the fused input data, where the sequential information can also be stacked as a matrix for each sample instead of a 1D concatenated vector (Stahlschmidt *et al*. 2022). For instance, in multi-omics studies, SNPs data are usually used simultaneously with DNA methylation data (Kang *et al*. 2022), and as a toy example with 2 modalities, we could be only interested in specific loci, and take into account the number of copies of the minor allele and whether or not there is an attached methyl group in those specific positions. So, we could merge these data from different channels as a 2×p matrix received by a convolutional layer, where each row contains data for a single modality and every column contains information for a particular locus. This approach also allows the NN model to not distinguish between features from different modalities and cross-modality and within-modality correlations are learned simultaneously at a low level of abstraction, that is, the model deals with raw or less processed data features, focusing on the specific and detailed attributes of the data. This means the model simultaneously learns detailed relationships and patterns within and between different types of data without simplifying or generalizing them too much (Pérez-Enciso and Zingaretti 2019). A particular approach can be found in the study by Wan *et al*. (2013), where genomic (SNPs) and transcriptomic (gene regulation) data, arranged in matrices are transposed and multiplied; then, the resulting matrix feeds the NN model, and this approach allows to leverage cross-modality correlations, even after the learning process.

On the other hand, the early data fusion strategy may present limitations when modalities have heterogeneous input dimensions or when they have different levels of relevance for the task in question. For example, in PB programs, usually in addition to genomic data, information with different structures is available, for example, in Bhadra *et al*. (2023), information provided by Unmanned Aerial Vehicle (UAV) images is leveraged by a 3D convolutional NN to predict soybean crop yield. In this case, this additional modality does not share structural similarities with genomic data, which usually are available as vectors containing the number of copies of the minor allele for the SNPs under study. Therefore, in situations like this, using an early fusion approach could not be the best choice. From experimental observations, it is recommended to resort to early fusion when the different modalities are of a similar nature and share relevant complementary information, and to use other strategies, when the modalities at hand have weak correlation. The practitioner should be careful when determining whether or not 2 modalities are correlated under the warning that choosing a fusion strategy blindly for 2 essentially different modalities at an early stage has a high risk of learning fake cross-modality correlations and interactions (Neverova *et al*. 2014).

In some cases, additional techniques such as transformations for specific modalities or attention mechanisms can be used to handle the discrepancies and use the multimodal information in the correct way. Attention mechanisms are a key innovation in the field of AI, particularly in natural language processing and computer vision. They allow models to focus on the most relevant parts of the input data when making predictions. Attention mechanisms mimic the human ability to focus on certain parts of an input while processing information. In AI, attention mechanisms enable models to dynamically highlight the most important elements in a sequence of data when making predictions. By focusing on the most relevant parts of the input, attention mechanisms significantly enhance the performance of MLML models, making them more accurate and efficient in handling complex tasks.

Raw data dimensionality can be reduced using encoders, jointly or separately for each modality, which increases the number of parameters to be trained. When the input modalities are in different dimensions for any type of data fusion, for instance, when data represented in 1D are combined with 2D or 3D image data, then high-level image features need to be extracted as a 1D vector before fusing with another 1D modality (Huang *et al*. 2020). Another point to consider is that if the number of samples in the training data is small compared to the number of features—which is usual in genomic information—the number of parameters in the model could be too high, increasing the computational cost. Furthermore, combining all modalities at an early stage of the model implies a high number of synaptic connections, which translates into an increase of computational resources and training time.

## Intermediate data fusion

This strategy, also known as joint fusion, helps merge modalities at different depths, learning latent feature representations within the model before fusing them into a common layer (Fig. 3).

Unlike early fusion, in this approach, latent feature representation learning is not separated from the subsequent model. CNNs or LSTMs are typically used to learn latent features. Lower-dimensional feature representations are not required for all modalities, often limited to unstructured data. The model head can be an MLP to capture interactions between modalities or a classical statistical model, for example, a linear, a logistic, or a generalized additive model for interpretability (Akkus *et al*. 2023). One of the advantages of intermediate fusion is that the dimensional difference between input modalities can be handled by first applying sub-models for some individual modalities to make them reduce their dimension. However, if such a difference is large, reducing the dimensionality of the modalities with higher dimensions too much could lead to a considerable loss of information (Stahlschmidt *et al*. 2022).

Another advantage of this fusion approach is the possibility of presenting flexible selection when finding the optimal number of layers and sequence to fuse marginal representations, as well as the potential to better reflect the true relationships between modalities, leading to the discovery of more meaningful joint and marginal latent factors. Marginal latent factor refers to the hidden variables or representations that capture the essential characteristics or patterns within each individual modality separately. These factors are derived from the data of each modality in isolation, without considering the information from other modalities, while joint latent factor refers to the hidden variables or representations that capture the combined information from multiple modalities. These factors are derived by integrating or fusing the marginal latent factors from each modality, allowing the model to learn and represent the relationships and interactions between different types of data. DL architectures are well-suited for intermediate fusion as they enable an easy connection of marginal representations to a shared layer, facilitating the correspondence of hierarchical representations to real-world phenomena (Stahlschmidt *et al*. 2022). Using this strategy allows distinct data features to create a more expressive representation, leveraging the strengths of each type of data. For example, features from Red, Green, and Blue (RGB) images and skeletal sequences can be fused and their separate advantages exploited simultaneously (Boulahia *et al*. 2021).

In some cases, not all modalities are available for every individual in the dataset. To tackle this problem, Thung *et al*. (2017) proposed a multitask network that effectively learns from data with missing modalities. This is achieved by assigning unimodal input branches and task-specific output branches, meaning predictive tasks are designated according to the corresponding available modality. Each task reflects the availability of one or more modalities. However, a limitation of this approach is that it is designed specifically for multitask predictions. In the context of genomic prediction, a multitrait prediction model where traits are the output-specific task can be reformulated as a multitask network with multiple modalities as inputs.

## Late data fusion

Also known as ensemble fusion or decision-level fusion, this involves leveraging predictions from multiple models to make a final decision. Every modality has its own separate model which is trained individually, and the final decision is made by combining the outputs using an aggregation function, which can include averaging, majority voting, weighted voting, or a meta-classifier based on the predictions from each model (Fig. 4).

The choice of the aggregation function is typically empirical and varies based on the specific application and input modalities (Huang *et al*. 2020). It should be noted that the most straightforward method of combining decisions from individual sub-models is to take the average of their outputs. For classification purposes, a reasonable procedure could be averaging out the probabilities obtained from softmax functions for each class. This approach assumes an equal contribution from each sub-model, as there is no weighting of their outputs (Stahlschmidt *et al*. 2022). However, because late fusion operates on inferences and not the raw inputs, it is not effective when modeling signal-level interactions between modalities (Liu *et al*. 2018); indeed, this approach could be beneficial when data from different modalities have little or no correlation. The most obvious disadvantage of this fusion strategy is the loss of valuable information coming from interactions, or cross-correlations between modalities, which takes relevance for plant breeders, since it is well known that the variation of phenotypic traits depends on interactions between genetics and environment (Guo and Li, 2023). Another issue to take care of is that of assuming that all modalities possess the same predictive relevance for the target of interest. To address this issue, are weighted the outcomes of sub-models according to their resulting error, assigning more relevance to modalities with less predictive certainty.

Late data fusion in genomic prediction involves combining the outputs from different data modalities or models at a later stage, rather than integrating the data at the beginning. Each modality of data (e.g. genetic sequences, environmental factors, and phenotypic data) is processed independently by its respective model or branch. These models generate intermediate outputs specific to the modality and task before they are combined. Examples include predicting the likelihood of disease or traits based on genetic markers, assessing the influence of environmental conditions on genetic expression, and estimating observable traits like yield, height, or resistance to diseases.

The intermediate outputs (sub-outputs) from all models are then combined in a fusion layer. This layer integrates the information from all modalities to generate a final prediction. The fusion layer processes the combined information to produce a comprehensive prediction. This final output leverages the strengths of each modality to improve accuracy and robustness.

An example comprises genetic and phenotypic data utilized in genomic prediction in PB: (1) genetic data that is an aggregate score reflecting the genetic predisposition to certain traits or diseases and the frequencies of specific genetic variants that may contribute to trait variations, and (2) the environmental data should have predictions on how well a plant will perform in different climatic conditions. In summary, by using late data fusion, genomic prediction models can effectively combine diverse sources of data to make more accurate and comprehensive predictions, even when some data modalities are incomplete or missing.

Ensemble methods can be considered a form of data fusion. In essence, both ensemble methods and data fusion involve combining information from multiple sources to improve prediction accuracy or decision-making. However, they are used in slightly different contexts and have distinct characteristics. While ensemble methods are typically discussed in the context of combining multiple models, they align with the broader concept of data fusion when applied to models trained on different data sources. Both approaches aim to integrate diverse information to achieve more robust and accurate predictions.

### The choice of an appropriate fusion strategy in genomic prediction of PB

Based on what has been mentioned previously, the choice of a fusion approach depends on the nature of the different modalities, on complementarity, on the correlation between modalities, and to a lesser extent, on the computational cost associated with every approach. Here, we provide some comments to consider when selecting a fusion strategy and summarize some of their most remarkable features (Table 2).

**Table 2.** Summary of advantages and disadvantages of different data fusion strategies.

	Early fusion	Intermediate fusion	Late fusion
Description	Features from all modalities are merged with no distinction of which features come from which modality		Every modality is processed separately by its own sub-model and the individual outcomes are combined to get a single prediction
Pros	Use of cross-modality correlations and interactions They have lower computational complexity compared to other fusion strategies because the fusion occurs at the input level	Effectively balances the use of cross-modality and within-modality correlations and interactions, optimizing the number of parameters required. They have moderate to high computational complexity depending on the complexity of the fusion mechanism employed Robustness to missing modalities Flexibility to choose the level where specific modalities are fused Compared to early fusion, intermediate fusion may be more efficient in terms of capturing interactions between modalities while avoiding excessively high-dimensional input spaces More robust than early fusion to noisy or incomplete data due to fusion at intermediate layers	Easy computational implementation They have relatively lower computational complexity More robust to noisy or incomplete data than early fusion as each modality is processed independently
Cons	High computational cost due to a high number of connections Risk of learning fake cross-modality correlations High number of parameters and neural connections Less robust to noisy or incomplete data due to direct combination at the input level	Risk of loss of information from cross-modality correlations	Loss of information from potential interactions and cross-modality correlations Late fusion may require more training time compared to early fusion due to the separate processing of each modality

Although, in some cases, the correlation between modalities could be measured by conventional measures such as Pearson’s or Spearman's correlation coefficient, when the input data come with structural similarities, the nature of modalities should be kept in mind. For instance, Zhang *et al*. (2021) and Zhang *et al*. (2021) illustrate semantic cross-correlation with the example of an image described by text. In this case, it is expected that certain elements in the text will refer to specific elements in the image, making the correlation between modalities obvious and justifying the use of early fusion. Another example, in the field of survival analysis, is provided by Huang *et al*. (2019). Here, mRNA and miRNA data are initially processed by separate MLPs due to the explicit assumption that these 2 modalities have cooperative but independent effects on the hazard function. These modalities are then fused at an intermediate stage.

In some cases, certain features in one modality may be correlated with features in another modality, while other features are not. To address this, modalities can be divided into sub-modalities. For example, in the study by Kick *et al*. (2023), soil conditions, weather, and management conditions are all considered environmental factors. However, soil conditions are treated as a separate modality, and a sub-model is trained for soil conditions before the fusion process.

To help potential readers select the best fusion strategy for their specific tasks, we summarize the main features of each approach as follows.

It is important to point out that early fusion can indeed present challenges when dealing with modalities that have heterogeneous input dimensions or levels of relevance. Here are some techniques and approaches commonly employed in practice to address these challenges: (1) dimensionality reduction: use techniques like principal component analysis (PCA), autoencoders, or t-distributed Stochastic Neighbor Embedding (t-SNE) to reduce high-dimensional modalities to a common lower-dimensional space before fusion, (2) modality-specific processing: process each modality independently with modality-specific NN branches before fusion to learn optimized representations. Employ modality-specific attention mechanisms or adaptive fusion layers for dynamic contribution adjustment, (3) feature engineering: extract domain-specific features or apply transformation functions to align representations of heterogeneous modalities before fusion. Techniques like histogram equalization, scaling, normalization, or domain-specific transformations can help, (4) attention mechanisms: dynamically focus on relevant parts of each modality's input using attention mechanisms to emphasize informative regions while suppressing noise. Utilize modality-specific or cross-modal attention mechanisms for effective fusion, (5) ensemble methods: combine predictions from models trained on different modalities using techniques like averaging or weighted voting to leverage strengths and mitigate weaknesses. These strategies can be used individually or in combination to manage heterogeneous modalities effectively in early fusion scenarios.

Choosing an appropriate fusion strategy for plant genomic prediction involves considering the types of data available and the specific prediction tasks. Assume a plant breeder is working on predicting grain yield and disease resistance of a particular crop with access to 3 types of data genetic sequences of the crop. Traits such as plant height, leaf color, previous grain yield, soil quality, temperature, and rainfall data are observed. Separate models are used for (1) using genetic markers to predict traits like grain yield and disease resistance, (2) leveraging observed traits to make predictions about future yield and resistance, and (3) constructing a model that assesses how environmental conditions impact yield and disease resistance. The generated intermediate output will contain (1a) genetic risk scores from the genotypic data model, that produce an output score that indicate the genetic predisposition for high yield and disease resistance (2a) environmental impact scores: From the environmental data model, output scores that predict the impact of environmental factors on yield and disease resistance. Finally, combined the intermediate outputs (sub-outputs) from the 3 models in a fusion layer. This layer could be a simple ensemble method (e.g. weighted average) or a more complex NN that learns to optimally integrate the various outputs. The fusion layer processes the combined information to produce final predictions for yield and disease resistance.

### On tuning hyperparameters

In genomic prediction, as in other ML tasks, it is crucial to properly separate the data into training, validation (or tuning), and testing sets to ensure the model's reliability and generalizability. Like their unimodal analogs, MMDL models also include nonlearnable (hyperparameters) parameters. While learnable parameters (weights and biases) are learned during the training process, hyperparameters are set before the beginning of the learning process. Thus, hyperparameters (number of neurons in every hidden layer, number of hidden layers, kernel-order, stride, padding, or choice of activation function, etc.) are not learned by the model. Different hyperparameters can lead to considerable differences in performance. Furthermore, an excessive number of hyperparameters could result in overfitting, which translates into the loss of predictive power. Hence, such quantities would be selected meticulously, which in most cases is challenging but necessary. Sun *et al*. (2015) dedicate an article to discussing theoretical aspects of the number of hidden layers in neural networks and conclude that for neural networks with a limited number of hidden neurons, increasing arbitrarily, the number of layers could not be a good idea, since there is a clear tradeoff between its positive and negative impacts on test error, suggesting a loss of predictive power. Regarding the number of neurons in every hidden layer, in the study by Bengio 2012, 2012 it is suggested to include several nodes large enough in every layer, and it is remarked that using the same number of neurons in every layer, rather than a sequential decrease or increase of them, seems to be a better choice. However, the same paper claims that such advice is based on experimental results and every data set could require a specific choice for such hyperparameters.

Several frameworks have been proposed for tuning the optimum hyperparameters in DL models. Some of the most popular are grid search, random search, and Bayesian optimization (Supplemental Material).

### MMDL frameworks

MMDL models can be implemented using popular libraries such as Keras, which serves as the front-end, and TensorFlow, which acts as the back-end (Chollet and Allaire 2017). Both Keras and TensorFlow are known for their user-friendly interfaces. PyTorch and Chainer are also efficient options for implementing complex DL models (Tokui and Oono 2015; Team PC, 2017), including MMDL models. PyTorch was introduced to combine usability and speed in the same framework (Paszke *et al*. 2019) for DL tasks.

For PB applications, Keras (Chollet and Allaire 2017) in R or Python provides a user-friendly interface for DL implementation. However, a basic understanding of DL concepts is necessary, as specifications such as activation functions, loss functions, and metrics for validation sets must be provided manually, and the number of hidden layers must be chosen by the user. Open-source frameworks with user-friendly interfaces enable straightforward implementation of sophisticated DL models across various scientific domains, democratizing DL accessibility for professionals without extensive backgrounds in computer science or mathematics. This trend promotes wider adoption of MMDL by researchers in diverse fields.

For further details on DL frameworks, readers can refer to Chollet and Allaire (2017), Pérez-Enciso and Zingaretti (2019). Another increasingly popular Python module is Fastai (Howard and Gugger 2020), a high-level DL library built on PyTorch that simplifies the creation and training of complex models. Fastai offers prebuilt functions and classes for tasks such as data preprocessing, model creation, training, and assessment, making DL more accessible to inexperienced users and researchers.

More recently, MultiZoo (Liang *et al*. 2023) has been released in 2023. This comprehensive toolkit serves as a foundational codebase for multimodal algorithms, incorporating diverse methods for data preprocessing, data fusion strategies, optimization tasks, and training methodologies. MultiZoo is accompanied by MultiBench, an expansive benchmark comprising multiple datasets with up to 10 available modalities, facilitating the study and comparison of multimodal methods.

Sometimes, when implementing DL models, the lack of enough data is a limitation to obtaining the desired results. Thus, the use of pretrained DL models could be a valuable tool. This approach is known as transfer learning, which particularly in the context of MMDL, offers valuable opportunities to leverage knowledge gained from pretrained models in one modality to enhance performance in another modality within fusion strategies. Here is how transfer learning can be applied and its potential benefits: (1) knowledge transfer can be helpful to apply knowledge from pretrained models in one modality to initialize or fine-tune shared layers in multimodal architectures, (2) domain adaptation: employ techniques like adversarial learning or fine-tuning to adapt pretrained representations to target modalities, improving performance. For this reason, transfer learning is helpful to reduce reliance on large, labeled datasets in each modality by leveraging knowledge from pretrained models, and in general transfer learning enhances multimodal fusion by leveraging pretrained models, extracting features, adapting domains, and improving performance across diverse modalities. Illustrative examples can be found in the study by Wan *et al*. (2013) and Chandrashekar *et al*. (2023), where a model trained with complete data is used to imputation for incomplete data.

## Publications on MMDL applied to GS

Table 3 includes publications on applications of MMDL methods in GS or related fields, particularly focusing on studies involving molecular marker data (single nucleotide polymorphisms, SNPs) for PB purposes and comparisons of MMDL with unimodal DL models.

**Table 3.** Publications with actual or potential applications to genomic prediction.

Obs	Year	Authors	Crop(s)	Model	Modalities	Response	Compared to
5	2021	Shook *et al.*	Soybean	LSTM without attention and LSTM with attention	Genotypes (clusters of SNPs), maturity groups, and weather variables	Seed yield	SVR–RBF, Lasso, and data-driven model from USDA
6	2021	Danilevicz *et al.*	Maize	Tab-DNN and Sp-DNN combined	Genomic (SNPs), management practices, and multispectral images	Grain yield	Tab-DNN (unimodal), random forests, and XGBoost
7	2021	Måløy *et al.*	Barley	Plain performer, historical performer, and multimodal performer	Genomic (SNPs), and weather	Grain yield	BGLR-RKHS, CNN-MLP, and ResNet-MLP
8	2022	Zhao *et al.*	Simulated data	NN-MM (neural network-mixed model)	Genomic (SNPs), epigenomic, transcriptomic, and proteomic	Simulated phenotypes	Single-step
9	2022	Jácome-Galarza	Maize	CNN combined with LSTM	Multispectral images and field sensor data over time	Yield	Unimodal LSTM
11	2022	Sharma *et al.*	Maize	DeepG2P	Genomic (SNPs), environment, and management practices	Grain yield	GEBLUP, CNN-21, and AutoCGM
12	2022	Kaur *et al.*	Corn and soybean	CropYieldNet	Surface reflectance band, soil, and meteorological data	Yield	CNN [112], CNN + GaussP [112], and CNN + LSTM [113]
13	2023	Kick *et al.*	Maize	DNN-CO and DNN-SO	Genomic (SNPs), weather, soil, and management	Grain yield	Intercept, LM, GBLUP, K-NN, RNR, RF, SVR
14	2023	Montesinos-López *et al.*	Wheat	MLP and ResNet	Genomic (SNPs), NDVI (images), and year	Grain yield and thousand-grain weight	GBLUP, GBM and SVR
16	2023	Togninalli *et al.*	Wheat	Multimodal PheGeMIL	Genomic (SNPs), multispectral and images, and digital elevation models (DEMs)	Grain yield	Lasso, RF, and PheGeMIL for several combinations of modalities
17	2023	Chandrashekar *et al.*	Human traits	DeepGAMI	Genomic (SNPs) and Gene expression (transcriptomics)	Schizophrenia status, and Lung cancer stage	—

DT, decision trees; XGBoost, eXtreme Gradient Boosting; GRidge, adaptive group-regularized ridge regression; PLSDA, partial least squares discriminant analysis; sPLSDA, sparse partial least squares discriminant analysis; VCDN, view correlation discovery network; USDA, United States Department of Agriculture; LR, logistic regression; DA, discriminant analysis; DDA, diagonal discriminant analysis; NC, nearest centroid; PLS, partial least squares; GaussP, Gaussian process; LM, linear model; RNR, radius neighbors regressor.

Publications are arranged chronologically, detailing the crops or species where MMDL was applied, the specific MMDL model utilized, the predicted response variables (traits), and the comparative models used alongside MMDL. We have endeavored to include all relevant studies concerning MMDL applied to GS in PB. Additionally, other publications were selected based on their potential applicability of MMDL to GS, despite not explicitly using the term “multimodal data”?. These studies often refer to “multi-omics data,”? encompassing genomics, transcriptomics, proteomics, metabolomics, and phenomics (Mahmood *et al*. 2022). In the following summary table, we focus solely on the prediction performance of these DL models. Detailed implementations are provided in Supplementary Appendix C.

## Examples of the genomic prediction performance of MMDL methods in PB

MMDL frameworks excel at integrating diverse data types, including genomic, phenotypic, environmental, and pedigree information, by leveraging their ability to learn complex patterns across modalities. A hypothetical example of how MMDL could be applied effectively in the context of crop breeding by integrating genomic, phenotypic, environmental, and pedigree data is presented below. The data type is from (1) genomic where SNP markers represent the genetic variation among breeding lines, (2) phenotypic that observed traits like yield, plant height, and disease resistance are collected in different environments, (3) environmental variables such as temperature, rainfall, and soil type for each testing location, and (4) pedigree information that indicates the genetic connections among lines, providing a historical genetic context.

The MMDL framework uses data encoders where each individual NN encodes each data modality separately. The genomic encoder includes a deep neural network (DNN) for SNP data to capture genetic variation, the phenotypic encoder has another NN that encodes observed traits, focusing on patterns within the phenotypic data, the environmental encoder includes recurrent NN or attention mechanism that captures temporal and spatial patterns in environmental data, and finally, a pedigree encoder where a GNN or another specialized model captures the relationships between lines based on pedigree.

An intermediate data fusion strategy encoded representations from each modality combined in a fusion layer that learns complex interactions between genetic, phenotypic, environmental, and pedigree data. This fusion can involve concatenation, attention mechanisms, or more complex operations like tensor fusion to enhance the model's understanding of multimodal interactions. Finally, the prediction layer is used where he fused representation is passed through a final NN layer that predicts the target trait, such as yield performance, under specific environmental conditions.

The practical impact, in a real breeding program of this integrated approach, could significantly shorten the breeding cycle by providing more accurate early-stage predictions of high-yielding lines. Breeders can make informed decisions on which lines to advance, cross, or discard, ultimately enhancing the breeding program's efficiency and genetic gain. This example suggests how MMDL frameworks utilize their strength in multimodal learning to integrate diverse data types, capturing intricate patterns that drive superior predictive performance in complex biological systems like PB.

In summary, the advantages of MMDL over other traditional ML methods include (1) can capture intricate, nonlinear relationships between genetic markers and phenotypic traits, which traditional GBLUP models might miss due to their linear assumptions, (2) allows for the integration of multiple types of data (e.g. genomic, environmental, and phenotypic) simultaneously, leading to more comprehensive and accurate predictions, (3) MMDL models are well-suited for managing high-dimensional datasets, such as those generated by high-throughput sequencing technologies, enhancing prediction performance, (4) MMDL frameworks can adaptively learn and update their models based on new data, improving their predictive accuracy over time compared to static GBLUP models, and (5) they enhance feature extraction and transformation, enabling better representation of complex genetic information and environmental interactions.

Hereafter, we will highlight the main research efforts that apply MMDL approaches in PB. Our focus is on some of the key studies that integrate diverse data types to enhance the prediction of breeding values. While we strive to cover the most impactful and representative work in this rapidly evolving field, we acknowledge that the review may not capture every study due to the ongoing advancements and the breadth of research being conducted.

Shook *et al*. (2021) developed 2 LSTM-based models for crop yield prediction in soybean, namely, Stacked LSTM model (without the use of any attention mechanism) and Temporal Attention Model (using a temporal attention mechanism). The proposed methods were compared to SVR with radial basis function kernel (SVR–RBF), and least absolute shrinkage and selection operator (Lasso) regression (Supplementary Table 1). Three evaluation metrics were used: root mean square error (RMSE), MAE, and the coefficient of determination or *R*-squared ($R^{2}$) score. The proposed models surpassed the SVR–RBF and Lasso in terms of all the 3 metrics. Furthermore, the multimodal versions of those models showed similar or higher performance than their unimodal (only weather variables) in every case (Supplementary Table 1).

Danilevicz *et al*. (2021) introduced an MMDL designed to predict the performance of maize (*Zea mays*) during its early developmental stage, aiming to enhance the speed of crop breeding. They combine multispectral images, and 8 vegetation indices obtained from a UAV ∼60 d after sowing, across three consecutive growing seasons (2017–2019). By integrating management practices (seed stock, fertilizer use, and planting date) data and genotype information (2 parental lines) on the one hand, with tab-DNN (for tabular data) and multispectral (images) data with sp-DNN on the other hand, the proposed model aims to capture contextual factors that influence plant growth during the trial period. The individual performance of tab-DNN was compared against the RF and XGBoost models, where RF (with one-hot coding) seems to be the best model, along with RMSE, RMSE%, and *R*^2^ of 1.46 ± 0.08, 10.35 ± 0.54, and 0.55 ± 0.05, respectively, for the validation dataset, while the tab-DNN gave 1.67 ± 0.04, 11.87 ± 0.33, and 0.41 ± 0.02, respectively, for the same metrics (Supplementary Table 2a and b). However, when tab-DNN and sp-DNN were fused, their joint predictive power clearly surpassed that of separated models. In every case, the fusion of all modalities outperformed the separated approaches where the highest prediction performance was achieved by scaling the predictions from the tabular, spectral, and fusion modules using the weights defined during training.

Måløy *et al*. (2021) present a novel DL framework based on performers, introduced byChoromanski *et al*. (2020) by substituting the softmax self-attention mechanism in conventional transformers with a FAVOR + (Fast Attention Via positive Orthogonal Random features) mechanism. The aim of this approach was to effectively predict crop yield using SNPs and weather data. They compare the following models in the context of predicting barley yields across 8 different locations in Norway for the years 2017 and 2018: CNN for SNPs combined with MLP for weather data; Resnet for SNPs combined with MLP for weather data; Performer for SNPs combined with MLP for weather data (Plain Performer); Performer for SNPs combined with Performer for weather data (Historical Performer); Multimodal Performer for both modalities (genotype and weather) and Bayesian reproducing kernel Hilbert spaces generalized linear regression (BGLR-RKHS) only for SNPs data. The results of this study show that the performer-based models significantly outperform the traditional DL approaches, as well as the mentioned Bayesian method (Supplementary Table 3).

Zhao *et al*. (2022) proposed a neural network- mixed model (NN-MM) for integrating and analyzing multimodal data in the context of genomic prediction. This method extends linear mixed models (GBLUP) to multilayer neural networks for genomic prediction by integrating intermediate omics features: epigenomics, transcriptomics, and proteomics. NN-MM captures the cohesive network of multilayer regulations from genotypes to intermediate omics features and ultimately to phenotypes, effectively modeling nonlinear relationships between these features and observable traits. The model encodes the impact of genotypes on intermediate omics features and the regulatory influence of these features on downstream phenotypes. Compared to the single-step mixed effects model by Christensen *et al*. (2021), NN-MM demonstrated superior prediction accuracy, especially for individuals with missing omics data, as shown in Supplementary Table 4. Including individuals without omics data in both NN-MM and the single-step approach was more effective than excluding them.

Jácome-Galarza (2022) introduces a DL approach to predict corn yield in Ecuadorian croplands by combining multispectral images from Google Earth Engine and field sensor data (humidity, temperature, and soil status). Although this study does not include genomic data, it is relevant because multispectral images can enhance genomic prediction in multimodal tasks, as suggested by Danilevicz *et al*. (2022). The proposed model integrates a CNN for multispectral images and LSTM network for temporal sensor data from 2002 to 2020. The combined modalities feed into an MLP. The model achieved an MSE of 0.0454 and an MAE of 0.1958, improving yield prediction compared to a unimodal LSTM model, which had an MSE of 0.0480 and an MAE of 0.1996.

Wang and Chen (2022) proposed the DeepPerVar multimodal model, which utilizes genomic and phenotypic data to predict quantitative genome-wide epigenetic signals and uncover canonical motifs regulating gene expression, especially in genes associated with Alzheimer's disease (AD). DeepPerVar was compared to DeepSea, ExPecto, and DeepFIGV using LD score regression and categorized 220 cell-type-specific annotations into 10 groups, including adrenal/pancreas, central nervous system, cardiovascular, connective/bone, digestive, immune/blood, kidney, liver, skeletal muscle, and others. The study concludes that DeepPerVar effectively predicts genome-wide epigenetic signals and excels in identifying crucial genomic regions linked to AD. It successfully identifies canonical and AD-associated motifs, prioritizing potential causal variants within a GWAS locus. Notably, DeepPerVar outperformed DeepFIGV and ExPecto in heritability partitioning analysis.

Sharma *et al*. (2022) introduce Deep Learning Genotype-to-Phenotype (DeepG2P), a multimodal model integrating genomic, environmental, and management data to predict phenotypes. Using data from the G2F initiative, DeepG2P outperformed other models in Pearson correlation coefficient (PCC) for environmental splits and showed the lowest variance in RMSE. For hybrid splits, DeepG2P had lower variance, though CNN-21 and GEBLUP had better PCCs, and AutoCGM had better RMSE performance. DeepG2P excelled in handling new locations. An ablation study demonstrated the importance of genomic data and GxE cross-attention modules, with significant performance drops when these were removed.

Kaur *et al*. (2022) introduce CropYieldNet (CYN), a MMDL model for crop yield prediction. CYN consists of 3 modules: (1) Surface Reflectance Encoder: A 1D CNN capturing spatial patterns in surface reflectance data while preserving temporal patterns. (2) Soil Data Encoder: A CNN learning pixel intensity information for individual soil attributes. (3) Core Temporal Module: A series of bidirectional LSTMs leveraging temporal patterns in surface reflectance and meteorological data to predict yield. Although it does not include genomic data, the modalities used have potential applications in PB programs. The model was assessed using data from corn and soybean crops in the USA and India, and compared against a typical CNN, a CNN augmented with a Gaussian process (CNN + GP), and a multilevel CNN-LSTM model (CNN + LSTM). The authors conclude that CYN outperforms these baseline models and excels in generalizing crops in unobserved geographies.

Kick *et al*. (2023) developed an MMDL model integrating genomic, weather, soil, and management data from the G2F initiative to analyze genotype, environment, and management effects on maize yield (Supplementary Table 5). The study compared DNNs optimized for individual modalities against linear fixed effects models (BLUP), K-nearest neighbors (K-NNs), radius neighbors regressor (RNR), RF, and support vector machine (SVM). The MMDL approach achieved a low RMSE (0.948) and normalized root mean square error (NRMSE) (14.554%), closely matching the BLUP model (RMSE 0.937 and NRMSE 14.388%) and showed greater consistency across replicates. While SVM performed best for soil data alone, most models excelled with weather/management data. Overall, MMDL models outperformed unimodal models but did not always surpass traditional approaches.

Montesinos-López *et al*. (2023) applied a MMDL framework to analyze 2 wheat datasets incorporating genomic data, Normalized Difference Vegetation Index (NDVI), and years as modalities. They compared their approach with GBLUP, gradient boosting machine (GBM), and SVR models using various combinations of these modalities. The MMDL model demonstrated superior performance in predicting traits like yield (YLD), thousand-grain weight (TGW), and NDVI averages and dates compared to the baseline models. Evaluation metrics such as nRMSE and PCC favored the MMDL approach. For instance, when predicting TGW using genomic and year data, GBLUP achieved an nRMSE of 0.0663 ± 0.0089, slightly better than MMDL's 0.0744 ± 0.0081 (Supplementary Table 6). Similarly, for averaged NDVIs, GBLUP had an nRMSE of 0.0674 ± 0.0094 compared to MMDL's 0.0695 ± 0.0099, with a marginal advantage in PCC for GBLUP. However, integrating NDVI data showed mixed results in PCC improvement in the multimodal approach.

Togninalli *et al*. (2023) introduce Phenotype–Genotype Mutual Information Learning (PheGeMIL), a DL framework incorporating multichannel temporal inputs to enhance grain yield prediction using UAV images. The model leverages attention mechanisms to improve interpretability and considers four data modalities: multispectral images, thermal images, digital elevation models (DEMs), and genetic variants (SNPs). PheGeMIL significantly outperforms benchmarks like Standard, Lasso, Ridge, Elastic Net regression, Gradient Boosting, and RF. With the inclusion of genotypic information, the model achieves a PCC of 0.754 ± 0.024, compared to 0.707 ± 0.027 for RF and 0.708 ± 0.029 for Lasso. The integration of all data channels results in a PCC of 0.767 ± 0.019, showcasing the model's superior predictive capability.

As previously mentioned, MMDL frameworks excel at integrating different types of data, such as genomic, phenotypic, environmental, and pedigree information. This integration enables the capture of complex relationships between different data modalities that are often overlooked by traditional models which primarily focus on genomic data alone. MMDL can learn hierarchical feature representations, making it particularly suitable for capturing interactions between genomic markers and environmental factors (Montesinos-Lopez, Montesinos-López, Crossa, *et al*. 2018; Montesinos-Lopez, Montesinos-López, Gianola, *et al*., 2018; Chen *et al* 2021).

MMDL methods are modeling nonlinear and high-order interactions among predictors and in crop breeding, for example, MMDL can fuse SNP genetic data, phenotypic traits (e.g. yield and height), environmental variables (such as temperature and soil type), phenomics (high-throughput phenotype data), and pedigree information to enhance breeding predictions. MMDL employs specialized neural networks tailored to each data type: deep neural networks for genomic data, recurrent or attention mechanisms for environmental data, and graph neural networks for pedigree data. These networks are combined in a fusion layer to capture complex interactions before making predictions through a final NN layer.

## A simple MMDL example with python code

An example of an MMDL model incorporating environmental (Env) and genotypic effects (GID) (Env + GID) can be found in the study by Montesinos-López *et al*. (2024). In this study, 4,464 wheat lines were tested across 4 environments during the 2021/2022 crop season at the Norman E. Borlaug Experiment Station in Ciudad Obregón, Sonora, Mexico (27°20′N, 109°54′W). The phenotypic data included measurements for 5 traits: Yield, Germination, Heading, Height, and Maturity. The genotypic information comprised 18,239 SNP markers, with raw data filtered based on a minor allele frequency (MAF) cutoff of < 5% and a missing data threshold of < 50%. More detailed information about the experimental data can be found in the study by Montesinos-López *et al*. (2023).

## For consideration regarding MMDL methods

The most evident advantage of MMDL models is their ability to harness valuable information that would otherwise be underutilized. As demonstrated in the previous section, this approach often outperforms unimodal DL models and other commonly used ML techniques such as RF, SVM, Lasso, and XGBoost. Moreover, there exists a plethora of architectures that can be tailored to the specific nature of the data under consideration. For example, if incorporating image data alongside genomic information, a CNN architecture can effectively handle this additional complexity. Alternatively, for environmental data, an MLP could seamlessly integrate into the model. In cases involving temporal experimental data, RNNs like LSTMs, as demonstrated by Shook *et al*. (2021) and Wang and Chen (2022), or ResNets to mitigate the GV issue, may be employed. Despite the computational demands inherent in DL methods, numerous DL frameworks have been developed to optimize these resources efficiently. Given the increasing adoption of MMDL frameworks over the past decade, substantial improvements in prediction performance can be expected by integrating all available modalities effectively.

As noted earlier, MMDL methods have demonstrated clear superiority in GS and PB contexts. However, their application in these fields is relatively nascent, resulting in limited available literature on the subject. One common challenge in MMDL applications is selecting an optimal data fusion strategy, which must be carefully chosen based on the nature of the modalities involved and their potential interactions.

Furthermore, computational costs must be considered, especially in genomic prediction tasks where data are typically high-dimensional, leading to potentially lengthy runtimes due to the large number of parameters involved. As highlighted by Wang *et al*. (2023), the choice of model can significantly impact runtime performance, necessitating a judicious selection of model architecture.

In some instances, studies such as Kick *et al*. (2023) and Togninalli *et al*. (2023) have shown no clear superiority of MMDL over unimodal DL in terms of prediction accuracy. Additionally, while including additional modalities may enhance predictive power, the computational expenses incurred may not always justify the benefits. Moreover, successful implementation of MMDL requires that data from each modality be available in both the training and testing datasets to ensure robust performance evaluation.

## Current trends and potential future directions of MMDL

Future trends in this field suggest a growing focus on addressing the interpretability challenge posed by the black-box nature of NN models, which can deter skeptical practitioners. Interpretable MMDL methods aim to enhance transparency and understanding of the decision-making processes involved in fusion strategies. These methods emphasize providing insights into how the model combines information from multiple modalities to make predictions or classifications.

For example, attention mechanisms highlight the relative importance of different modalities or features during the fusion process, enabling users to discern which inputs contribute most significantly to the model's decisions. Techniques like feature visualization or saliency mapping further aid in understanding by visually representing input data and corresponding model activations, facilitating the identification of key features driving decision-making. By offering more interpretable insights into how multimodal data are integrated and utilized, these methods increase transparency, trust, and understanding—critical for real-world applications in healthcare, finance, and other domains where explainable decision-making processes are essential.

In the realm of genomics, current and future trends in MMDL are shaped by the integration of environmental information, phenomics data, and other omics data. The focus is on achieving robust prediction accuracy to ensure the successful implementation of GS methodologies in breeding programs. DL architectures are increasingly adopted to enhance the efficiency and efficacy of GS. By embracing these advancements and exploring new frontiers, MMDL holds the potential to revolutionize genomics research by significantly improving prediction capabilities.

Also, transfer learning, particularly in the context of MMDL. Here's how transfer learning can be applied and its potential benefits: (1) knowledge transfer can be helpful to apply knowledge from pretrained models in one modality to initialize or fine-tune shared layers in multimodal architectures, (2) domain adaptation: employ techniques like adversarial learning or fine-tuning to adapt pretrained representations to target modalities, improving performance. For this reason, transfer learning is helpful in reducing reliance on large, labeled datasets in each modality by leveraging knowledge from pretrained models, and in general, transfer learning enhances multimodal fusion by leveraging pretrained models, extracting features, adapting domains, and improving performance across diverse modalities.

Finally, we need to be aware that implementing MMDL incurs additional costs compared to unimodal methods, including the need for more extensive data acquisition and preprocessing from multiple modalities, increased model complexity and parameter tuning, greater computational resources for both training and inference, and higher storage requirements for large datasets and complex models. Additionally, integrating and maintaining multimodal systems demands more development, testing, and ongoing updates, while also requiring specialized expertise across various domains, leading to increased personnel and training expenses.

## Conclusions

MMDL holds potential for applications across various fields that require leveraging diverse data types for prediction tasks, such as GS. Like its unimodal counterpart, successful implementation relies heavily on the availability of high-quality and sufficiently large training datasets. While publications on the use of MMDL for GS suggest superior prediction power compared to conventional unimodal DL models, it is essential to acknowledge that multimodal techniques require substantial computational resources. Evidence often indicates that multimodal algorithms excel in capturing interactions between different data modalities more effectively than unimodal DL models and other ML methods. They are also adept at integrating data from diverse sources across different stages of the training process.

MMDL algorithms offer significant potential for enhancing prediction accuracy through specialized architectures and data fusion strategies tailored to specific data types in PB programs and similar contexts where genomic prediction is pivotal. Researchers intending to employ multimodal DL in GS must exercise caution, as with unimodal DL, particularly when working with small training-testing datasets. Careful selection of hyperparameters is crucial for optimal performance. Given the current global demographic trends and the demonstrated predictive superiority of MMDL techniques over unimodal DL methods and traditional statistical ML techniques, it is prudent to prioritize the exploration and adoption of multimodal tools for addressing PB challenges amidst a growing world population and increasing food demands.

## Supplementary Material

iyae161_Supplementary_Data

## Data Availability

The Python code to implement and evaluate the model's prediction performance for the heading trait using a 5-fold cross-validation strategy is available through the following link: https://github.com/osval78/Multimodal_Genetics_Example. Further details on how to use the Python codes are found in the Supplementary material section. Supplemental material available at GENETICS online.
