# Correction to: A review of multimodal deep learning methods for genomic-enabled prediction in plant breeding

**DOI:** 10.1093/genetics/iyae200

**Published:** 2024-12-20

This is a correction to: Osval A Montesinos-López, Moises Chavira-Flores, Kismiantini, Leo Crespo-Herrera, Carolina Saint Piere, HuiHui Li, Roberto Fritsche-Neto, Khalid Al-Nowibet, Abelardo Montesinos-López, José Crossa, A review of multimodal deep learning methods for genomic-enabled prediction in plant breeding, *Genetics*, 2024, iyae161, https://doi.org/10.1093/genetics/iyae161

In the originally published version of this manuscript, author Kismiantini’s name was misspelled.

This error has been corrected.

