# Supplementary material for: A review of multimodal deep learning methods for genomic-enabled prediction in plant breeding
Source: Genetics. 2024 Nov 5;228(4):iyae161. doi: 10.1093/genetics/iyae161 (PMC11631469; doi:10.1093/genetics/iyae161)
Supplement: iyae161_Supplementary_Data [file iyae161_supplementary_data.docx]

**SUPPLEMENTAL MATERIAL**

### **Multi-Layer Perceptron’s**

A multilayer perceptron (MLP), also known as a fully connected neural network (FCNN) or feedforward neural network, is the most representative neural network architecture among DL models; and is regarded as the quintessential DL model (Akkuset al., 2023). Its main feature is that every neuron (or unit) in a layer is connected to every neuron in the next layer (see **Figure S1**) and its layers are called fully connected layers. The layers between input signals and output(s) are known as hidden layers.

As mentioned above, the synaptic connections between neurons have associated weights, which are adjusted during a training process in which the network's performance is optimized using an adequate loss function. The purpose of the fully connected layers in an MLP is to transform the input data through a series of non-linear transformations, known as activation functions, enabling the network to learn and extract relevant features from the data. These learned features are then used to make predictions or classifications in the output layer. We can formulate the MLP model from the less sophisticated model in equation (1) as follows. In the single artificial neuron model in (1), we aim to predict $\boldsymbol{y}$ from ${\boldsymbol{x}\mathbf{=(}\boldsymbol{x}}_{\mathbf{1}}\mathbf{,...,}x_{m}\mathbf{)}^{\boldsymbol{T}}$ as $\boldsymbol{y}\mathbf{=}\boldsymbol{\varphi}\mathbf{(}\boldsymbol{w}^{\boldsymbol{T}}\boldsymbol{x}\mathbf{+}\boldsymbol{b}\mathbf{)}$**,** but we could be interested in predicting not only an output y but also a set of outputs $\boldsymbol{z}_{\boldsymbol{1}}$**,…,** $\boldsymbol{z}_{\boldsymbol{k}}$, so we would need k individual models like that of (1), each one with its own parameters such that

$$\boldsymbol{z}_{\boldsymbol{j}}\mathbf{=}\boldsymbol{\varphi}\mathbf{(}\sum_{i=1}^{m} w_{i,j}x_{i}+b_{k}\mathbf{)=}\boldsymbol{\varphi}\mathbf{(}{\boldsymbol{w}_{\boldsymbol{j}}}^{\boldsymbol{T}}\boldsymbol{x}\mathbf{+}\boldsymbol{b}_{\boldsymbol{j}}\mathbf{)}$$

for $j=1,...,k$, where $\boldsymbol{\varphi}$ is a common activation function for all $\boldsymbol{z}_{\boldsymbol{j}}$. Thus, we have a set of outputs $\boldsymbol{z}_{\mathbf{1}}\mathbf{,...,}\boldsymbol{z}_{\boldsymbol{k}}$. Now we could use these new variables as input signals to make a definitive prediction for $y$ as

$$\boldsymbol{y}\mathbf{=}\boldsymbol{\psi}\mathbf{(}\sum_{\boldsymbol{j}\mathbf{=1}}^{\boldsymbol{k}} \boldsymbol{v}_{\boldsymbol{j}}\boldsymbol{z}_{\boldsymbol{j}}\mathbf{+}\boldsymbol{b}\mathbf{)}$$

Thus, we have constructed a MLP with a single hidden layer consisting of the neurons, or nodes, associated to $\boldsymbol{z}_{\mathbf{1}}\mathbf{, ... ,}\boldsymbol{z}_{\boldsymbol{k}}$, an input layer with neurons associated to $x_{1}, ... ,x_{m}$, and

an output layer with a single neuron associated to $\boldsymbol{y}$**.** We could stop here or create a new intermediate layer between the input signals $\boldsymbol{x}$ and the output $\boldsymbol{y}$. The number $L$ of hidden layers in a MLP neural network is called the depth. The choice of $L$, and the number of neurons in every layer should be tuned in accordance with every problem, keeping in mind that such selection should be computationally optimal.

Its relative simplicity has made it popular in bioinformatics. Notably, several studies have demonstrated the application of this architecture in genomic prediction, including Montesinos-López et al. (2018), Montesinos-López, O. A. et al. (2018), Pérez-Rodríguez et al. (2020), and Sandhu et al. (2021). For instance, Montesinos-López, A. et al. (2018) compare an MLP model to the well-known GBLUP statistical model, while Montesinos-López, O. A. et al. (2018) compare an MLP to a BMTME model (Montesinos-López, O. A. et al., 2016). In both cases, the MLP models outperform their statistical counterparts. In genomic prediction, the simplest approach is to assign a neuron in the input layer for each molecular marker under study.

### **Convolutional Neural Networks**

Convolutional Neural Networks (CNNs) are a type of artificial neural network widely used for image analysis (Sultana et al., 2018). They are essential in computer vision tasks, such as image classification and object detection. This is particularly relevant in the context of genomic selection (GS) applied to plant breeding, where images provided by Unmanned Aerial Vehicles (UAVs) and other remote sensing tools are used to observe plant phenotypes of interest (Gao et al., 2020). In some cases, genomic data can also be processed by CNNs, as demonstrated by Nazzicari and Biscarini (2022), where genomic data are arranged as kinship matrices, and by Ma et al. (2018), where input genomic vectors are convoluted.

A typical CNN consists of one or more blocks of convolutional and pooling layers, along with non-linear transformation (activation functions), followed by one or more fully connected layers (MLP) and a final output layer (see **Figure S2.a**). The convolutional layer is the fundamental building block of a CNN, where feature representations are learned from the input data. It comprises learnable convolution kernels or filters that compute the elements of distinct feature maps (see **Figure S2.b**) by performing elementwise multiplication of the kernel entries with selected regions of the input data and summing all the terms.

A kernel can be understood as a grid of numbers, known as the weights of the kernel. Each unit in a feature map is connected to a receptive field in the preceding layer. A convolutional layer benefits from parameter sharing, which helps reduce the model's complexity (Ghosh et al., 2020). In the example of convolution depicted in **Figure S2.b**, no additional elements (rows or columns) are added to the input grid. However, occasionally, additional columns and rows are added around the original input grid to prevent information loss. This augmentation is known as padding. Also, in **Figure S2.b**, note that the kernel slides one cell at a time over the input grid until the convolution operation is complete, indicating a stride of 1. However, it is possible to perform convolution with other stride values.

Pooling layers, or sub-sampling layers, take small regions of the convolutional output as input and down-sample them to produce a single output (see **Figure S3**). Various sub-sampling techniques, such as max-pooling, min-pooling, average pooling, and tree pooling, help summarize and preserve the most relevant information from the feature maps while effectively reducing their dimensionality. Usually, after convolutional and pooling layers, this architecture includes a flattening stage, where multi-dimensional representations are transformed into one-dimensional vectors. For more details on CNNs, refer to Ghosh et al. (2020).

In Ma et al. (2018), this architecture was successfully applied to genomic prediction of traits in bread wheat. The model consisted of an input layer, a convolutional layer with eight filters of dimension 1x18 (vector filters), a max-pooling layer with a length and stride of 4, followed by two fully connected layers before the final single-neuron output layer. The first fully connected layer had 32 neurons, and the second had a single neuron. ReLU was used as the activation function in every case, and dropout was applied after the pooling layer for regularization. For all 𝑛 individuals in the collected data, the input layer receives a genotypic vector whose entries are 0, 1, or 2, representing the number of copies of the minor allele for all 𝑝 molecular markers under study. Thus, these vectors are 𝑝-dimensional. Other examples where a CNN is used for genomic prediction include Sandhu et al. (2021) and Wang et al. (2023), where the model proposed by Ma et al. (2018), along with two other CNN models, outperformed the RR-BLUP statistical model.

### **Recurrent Neural Networks**

Recurrent Neural Networks (RNNs) are a type of artificial neural network specifically designed to capture patterns in sequential data, such as handwriting, time series, or genomes, making them suitable for some genomic prediction tasks. Unlike MLPs and CNNs, which process input data in a single forward pass without cycles, RNNs have a feedback mechanism that allows them to process information sequentially by maintaining an internal memory or hidden state (Schmidt, 2019). Intuitively, an RNN can be thought of as an augmented MLP, where neurons in specific layers feed their outputs back into previous layers of the network (see **Figure S4**).

The structure of RNNs enables the network to retain information from previous inputs and use it to make predictions or decisions at each step. The hidden state acts as a memory, retaining information about previous inputs and influencing the processing of future inputs. The basic building block of an RNN is a recurrent layer, which contains recurrent units or cells. The most commonly used recurrent unit is the Long Short-Term Memory (LSTM) cell, first introduced by Hochreiter and Schmidhuber (1997). LSTMs are designed to mitigate the vanishing gradient problem by using input, forget, and output gates. These gates control the flow of information, deciding what to update, retain, or discard. Combined with sigmoid activation functions, they prevent gradients from vanishing during training, allowing LSTMs to maintain information over long sequences without degradation. The sequential nature of DNA makes this architecture suitable for genomics. For example, Li (2019) used an RNN model to classify centromeric satellites in DNA sequences, with input sequences of DNA using one-hot encoding for bases and output classes assigned to each analyzed base. A similar approach was used by Hausmann and Kurtz (2021), based on the model presented by Li (2019).

Although LSTM models have remarkable advantages over conventional RNNs, they are not without limitations. They are sensitive to hyperparameters, requiring careful optimization, which often needs to be done manually (Che et al., 2024). Another issue is the typically large number of recurrent synaptic connections between layers, which increases computational cost.

### **Residual Networks**

Residual neural networks, or simply residual networks (ResNets), proposed by He et al. (2015) for image recognition with a focus on CNNs, are designed to address the vanishing gradient problem. This problem occurs when the more layers a network has, the more challenging it becomes for gradients to flow backward during training, hindering convergence and leading to poor performance. ResNets tackle this issue through the concept of *residual blocks*, which contain skip connections or shortcuts that directly connect certain layers to later layers in the network. These shortcuts allow gradients to bypass several layers, facilitating the backward flow of information during training. As a result, residual blocks enable the creation of very deep neural networks while maintaining stable gradients throughout the training process.

The working of a residual block can be mathematically represented as follows. Consider a block of stacked layers in a neural network and let $x$ be the output vector of the previous layers. We can think of the transformation due to the learning stage of the stacked block of layers as an underlying mapping $H(x)$, which can be broken down as $H(x)=F(x)+x$, where $F(x):=H(x)-x$ is called residual function (see **Figure S5**).

The intuitive justification for using this mechanism is that, beyond a certain number of layers, gradients start to exhibit issues such as the vanishing problem mentioned above. When this threshold is reached, a residual block can be introduced into the model. The identity term

𝑥 in the residual function protects gradients from degeneration, allowing input information to bypass some layers without being affected by the activation functions in those layers. This enables the introduction of more layers into the model without losing trainability. Besides the identity function, other functions can be used depending on the specific problem.

A particular example is found in Jubair et al. (2021), where a residual fully connected neural network (RFCNN) was applied for predicting phenotypes from genotypes. The initial layer processes data from all molecular markers under study, creating a hidden vector representation. Batch normalization follows, standardizing the data, and ReLU activation introduces non-linearity. Residual connections skip intermediary layers, improving information flow. This process is repeated several times, culminating in the output layer, which predicts phenotypes. In this case, the residual network outperformed decision tree and linear regression models but not the RR-BLUP statistical model. Later, some examples of applications of this architecture in the context of multimodal genomic prediction will be presented.

### **Transformers**

This architecture was introduced by Vaswani et al. (2017) in the paper "Attention is All You Need." Transformers are highly effective at handling sequential data and have gained popularity in various natural language processing tasks, including machine translation, language understanding, and text generation. Unlike RNNs, which rely on recurrent connections, Transformers utilize self-attention mechanisms exclusively. This allows Transformers to process input sequences in parallel rather than sequentially, resulting in faster training and inference times. Self-attention mechanisms enable the model to weigh the importance of different elements in the input sequence, allowing it to focus on relevant parts while considering their dependencies. The attention block focuses on specific parts or elements of input data, assigning varying levels of importance or relevance to them during the learning or inference process. It typically involves three main components: a query, a set of key-value pairs, and a scoring or weighting function. The query represents the element to which attention is directed, while the key-value pairs represent the elements in the input data. The scoring function calculates a relevance score between the query and each key, determining the weights or attention coefficients assigned to the corresponding values (see **Figure S6**).

Two widely used types of attention mechanisms are scaled dot product attention and multi-head attention. Transformers typically consist of an encoder-decoder architecture. The encoder processes the input sequential data, while the decoder generates the output sequence. Both the encoder and decoder are composed of multiple layers of self-attention and feed-forward neural networks. The self-attention blocks capture dependencies within the input sequence, while the feed-forward layers apply non-linear transformations to the encoded representations. One notable feature of Transformers is their ability to handle long-range dependencies effectively. Unlike RNNs, Transformers do not suffer from the vanishing or exploding gradient problem over long sequences, making them well-suited for tasks involving long-distance dependencies.

Jubair et al. (2021) implemented a Transformer model called GPTransformer for genomic prediction. The attention mechanism in this model emphasizes the encoder module, which consists of an embedding layer and a multi-head self-attention layer, complemented by an MLP before the final output layer. The authors introduced a self-attention mechanism to compute the attention of all molecular markers with respect to a specific marker, leveraging the interaction between markers to achieve better phenotype predictions. On the other hand, although the model implemented by Hausmann and Kurtz (2021) is essentially a recurrent network, it uses an attention mechanism before the final layer to leverage information provided by the entire network.

### **Graph Neural Networks**

Zhou et al. (2020) reviewed this architecture of neural networks. Graph Neural Networks (GNNs) are designed to work with data represented as graphs. A graph consists of a set of nodes or vertices denoted by 𝑉 connected by a set of edges denoted by 𝐸, where each node can have associated features or attributes. The key idea behind GNNs is to leverage the structural information encoded in the graph to learn relationships between the nodes. GNNs can be thought of as a generalization of traditional neural networks, where instead of working with fixed-size vectors or sequences, they operate on data with graph structures. The basic building block of a GNN is the graph convolution operation, where each node aggregates information from its neighboring nodes through a weighted sum or a more sophisticated aggregation function (see **Figure S7**).

This allows the GNN to capture the local structure and dependencies in the graph. Other relevant blocks in GNNs include graph recurrent operators, skip connections, sampling, and pooling, analogous to operations in CNNs, but computed over a graph structure instead of a rectangular array. The process is typically repeated for multiple layers, allowing the GNN to capture information from nodes at different distances in the graph. GNNs have been successfully applied to a wide range of tasks involving data with graph structures, such as node classification, link prediction, graph classification, and graph generation. The success of GNNs is attributed to their ability to exploit the inherent graph structure and capture complex relationships between data points, making them a powerful tool for dealing with data in the form of graphs.

This architecture has special potential in genomics, where it is often necessary to study interactions between genes. For example, in Rhee et al. (2017), GNNs are applied to breast cancer subtype classification. In this study, genes are treated as vertices in a graph, with adjacencies based on their gene expression profiles. A series of convolutional and sampling operations, like those shown in **Figure S7**, are applied. The resulting graph is merged by a fully connected layer with the result from a mechanism proposed by the authors called the relationship network, which is essentially an MLP that deals with a set of selected edges. Finally, the results are processed by a softmax activation to obtain the final prediction.

### **Autoencoders**

Autoencoders were introduced by Rumelhart et al. (1986) and are primarily used for unsupervised learning and dimensionality reduction. They are designed to learn efficient representations or compressed encodings of input data, which are then used to reconstruct the original input data as accurately as possible. The architecture of an autoencoder consists of an encoder, a latent representation (or encoded data), and a decoder (Michelucci, 2022). The encoder maps input data to a lower-dimensional latent space representation, often referred to as a bottleneck. The decoder then reconstructs the original input data from this encoding (see **Figure S8**)**.**

An autoencoder aims to minimize the reconstruction error, thereby learning to capture the most important features or patterns of the input data in the encoded representation. They can be regarded as self-supervised models, meaning they do not require explicit labels for training. One popular type of autoencoder is the traditional autoencoder, which consists of fully connected layers. However, there are various types of autoencoders, each with its own variations and specific purposes. For instance, convolutional autoencoders are used for image data, while recurrent autoencoders are used for sequential data. Autoencoders are often used for data denoising, anomaly detection, feature extraction, and dimensionality reduction (Michelucci, 2022). They are also frequently used as a pretraining step or feature learning component in more complex models.

Although genomic prediction is a supervised learning task, autoencoder modules are often included in other complex models. For example, they are used in attention mechanisms in transformers. In other cases, only the encoder section is used as a dimensionality reduction module, as seen in Chiu et al. (2019) and Sharifi-Noghabi et al. (2019).

### **Dropout**, **Dropconnect and Batch Normalization**

Dropout was proposed by Srivastava et al. (2014) as a regularization method to mitigate overfitting, a common issue in neural network architectures due to their large number of parameters. The technique involves randomly dropping out units (hidden or visible) from the network during training, along with all their incoming and outgoing connections (see **Figure S9**).

The choice of which units to drop is random. In its simplest form, each unit is retained with a fixed probability 𝑝, typically set empirically, often around 𝑝=0.5, which has shown effective across various networks and tasks (Srivastava et al., 2014). Wan et al. (2013) introduced Dropconnect as another regularization method to reduce model parameters and mitigate overfitting. Unlike dropout, Dropconnect randomly deactivates some synaptic weights instead of entire neurons in the network.

In genomic applications discussed later in this article, variations of these methods are used not only for regularization but also to enhance the interpretability of neural network models (Chandrashekar et al., 2023; Nguyen et al., 2021). On the other hand, Batch Normalization involves normalizing a set of values by subtracting their mean and dividing by their standard deviation. This normalization can be applied to input values or values from hidden layers in a neural network. Batch normalization allows for higher learning rates, reducing training time, and can act as a form of regularization, sometimes eliminating the need for dropout in certain scenarios (Ioffe and Szegedy, 2015).


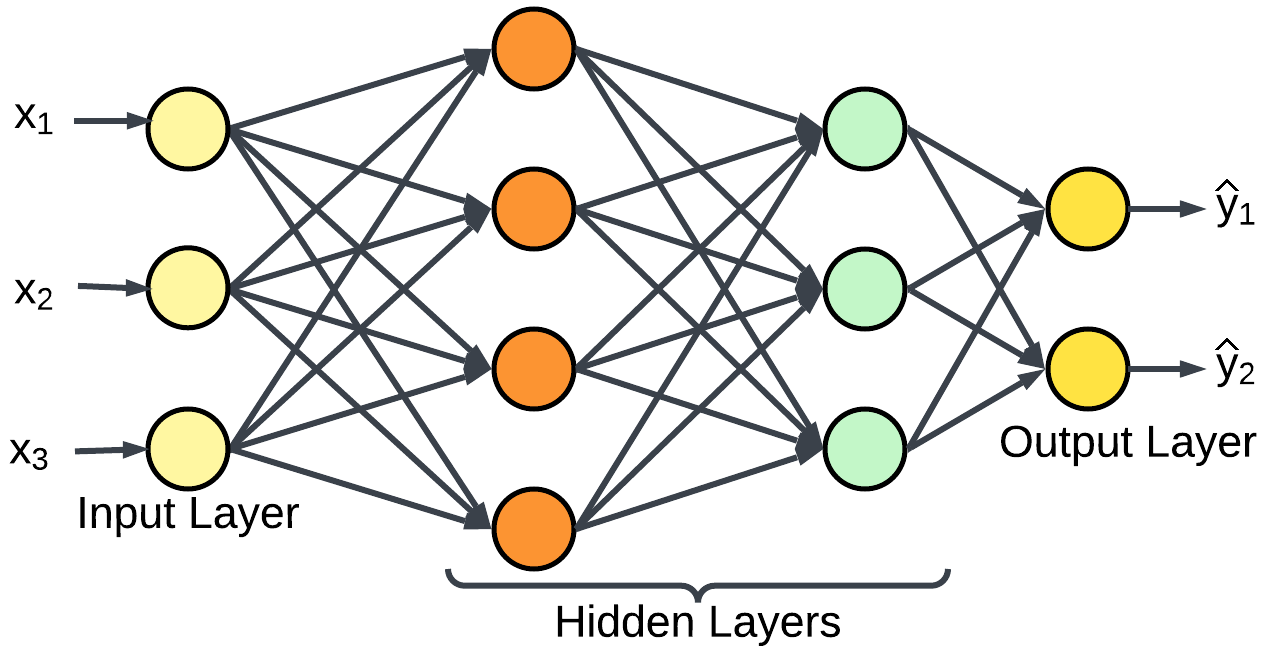


**Figure S1.** Example of an MLP with 3 inputs, $L=2$ hidden layers with $N_{1}=4$ and $N_{2}=3$ neurons respectively, and two outputs.


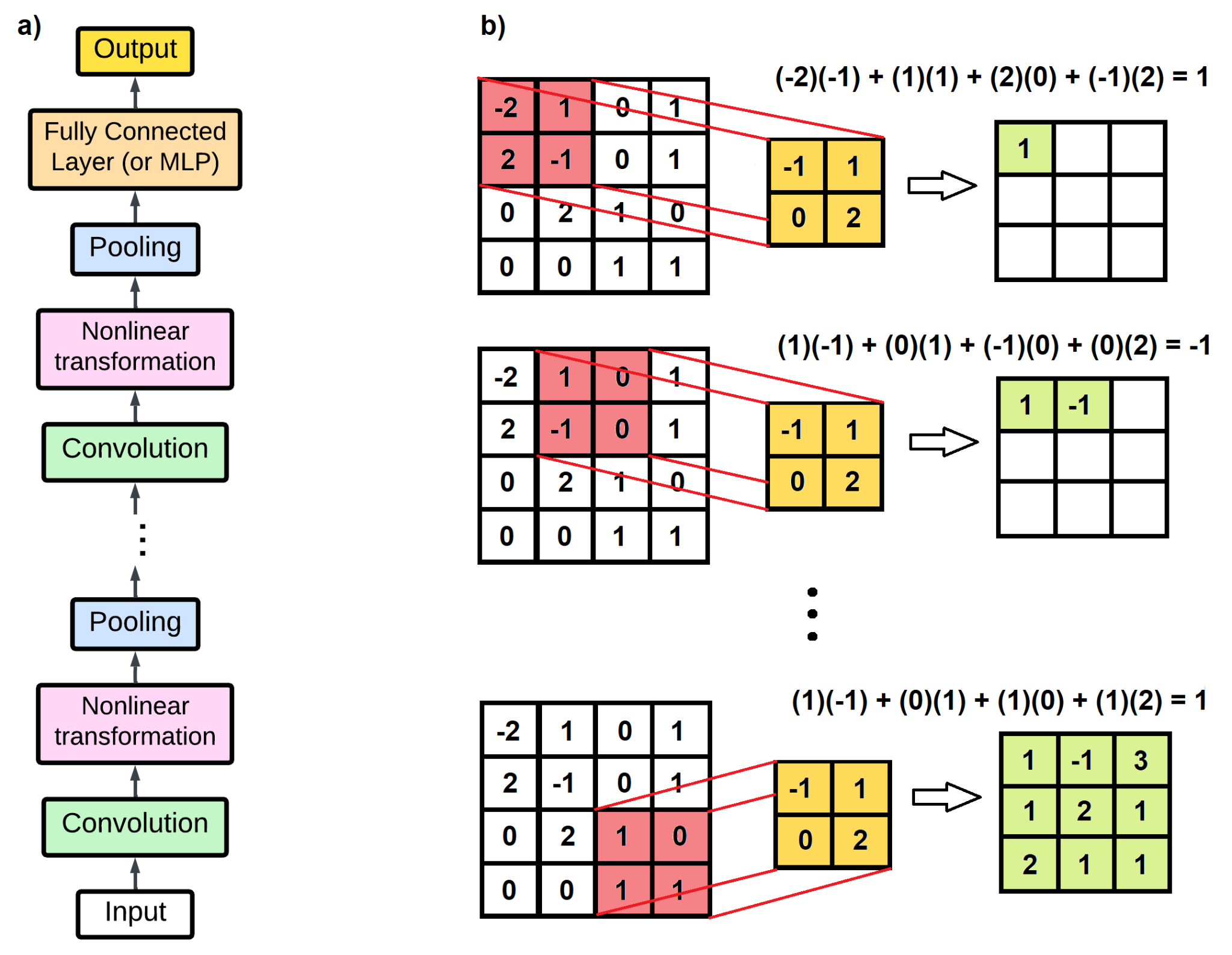


**Figure S2. a)** Structure of a typical CNN. Note that there are other possible order configurations of convolution, pooling, and nonlinear transformation**;** **b)** Example of a convolution operation, step by step. For brevity, only the two first and the last steps are shown. The yellow grid is a 2x2 kernel. The green grid on the lower right corner is the generated feature map resulting from convolution.


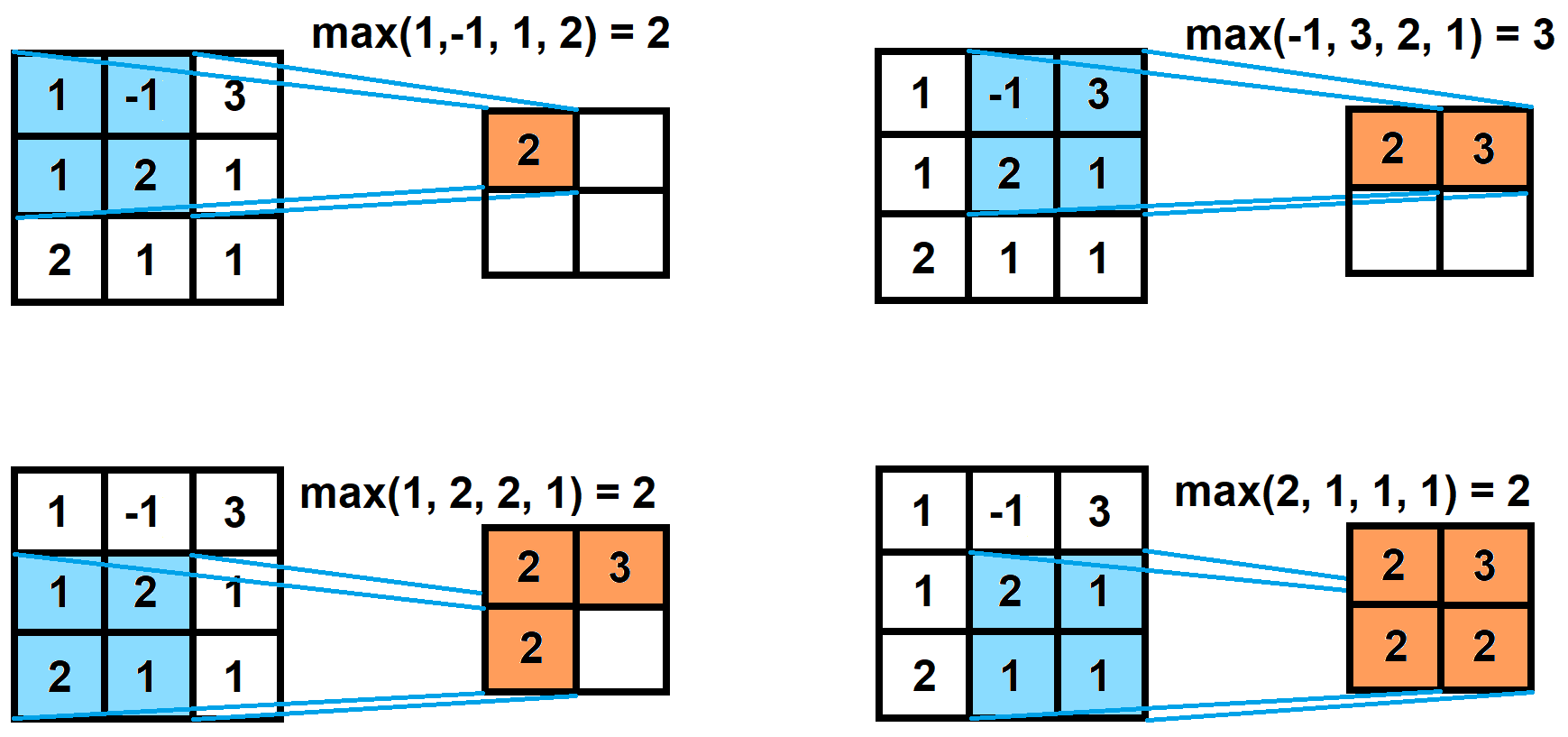


**Figure S3.** Example of pooling procedure, step by step, using max-pooling as a sub-sampling technique. The orange grid on the lower right corner is the final output of the pooling operation.


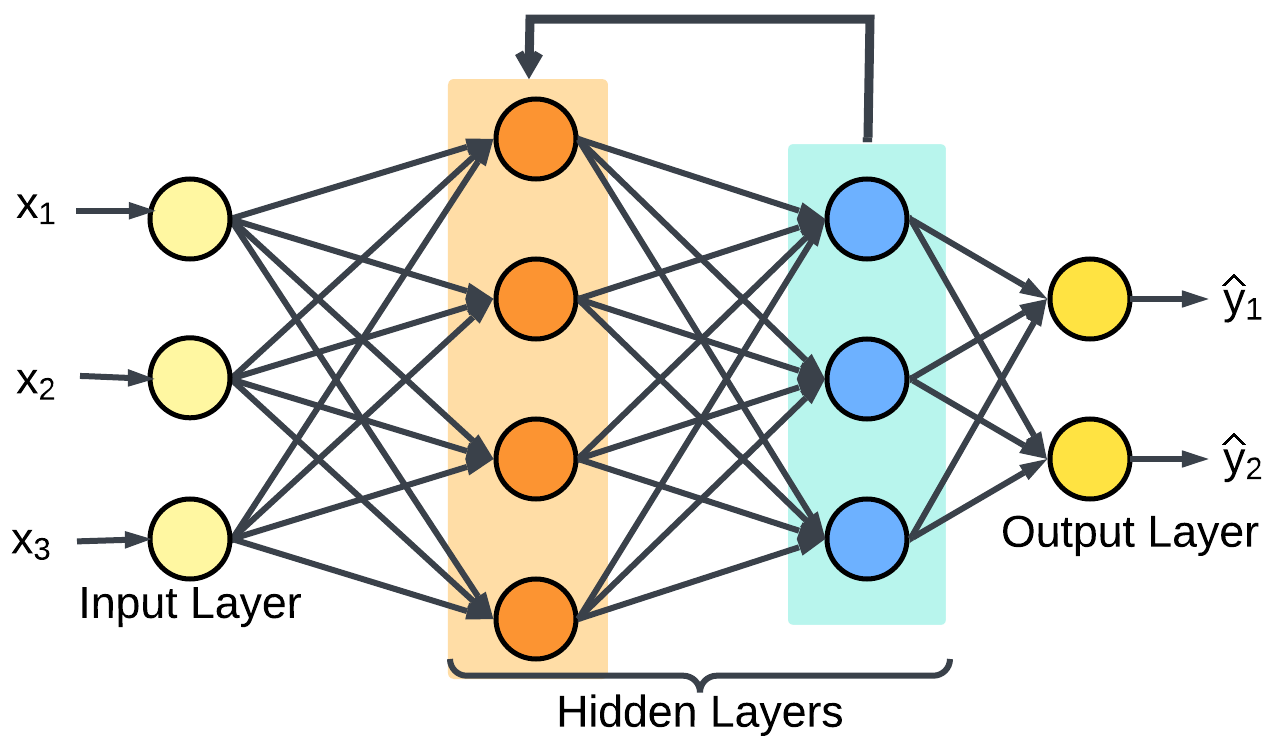


**Figure S4.** Example of recurrent neural network, based on **Figure S2**, where we have a MLP with 3 inputs, $L=2$ hidden layers with $N_{1}=4$ and $N_{2}=3$ neurons respectively, and two outputs. The arrow going from the blue-shaded layer to the orange-shaded layer indicates that the output of the second hidden layer feeds and serves as input for the previous layer.


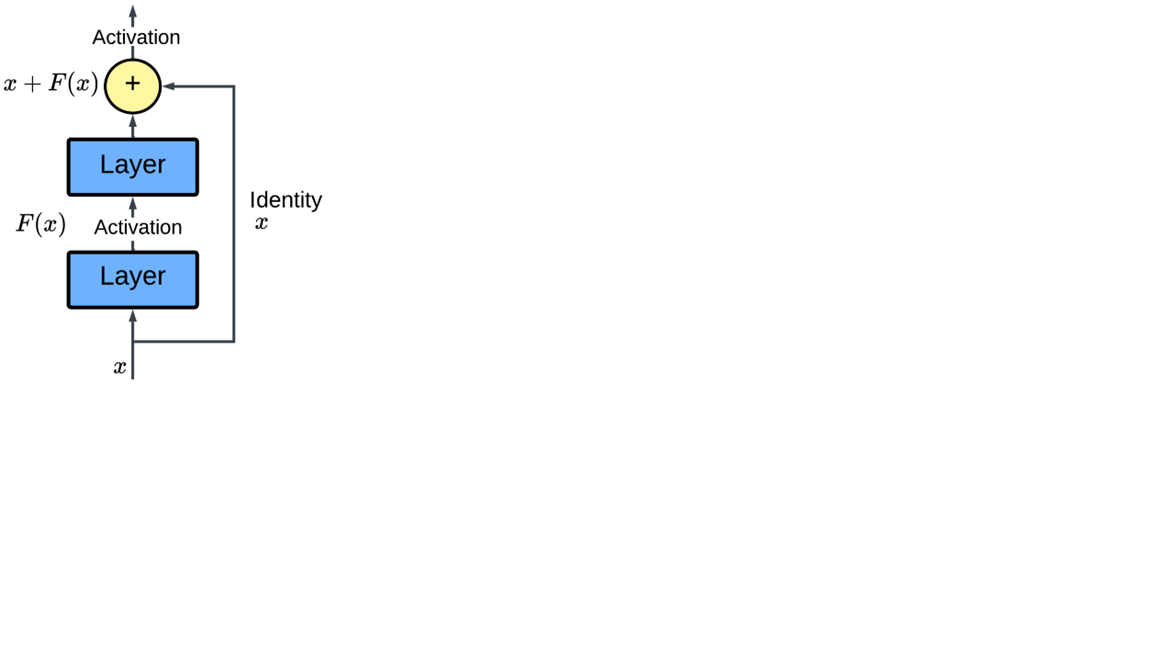


**Figure S5.** Representation of a residual block in a ResNet


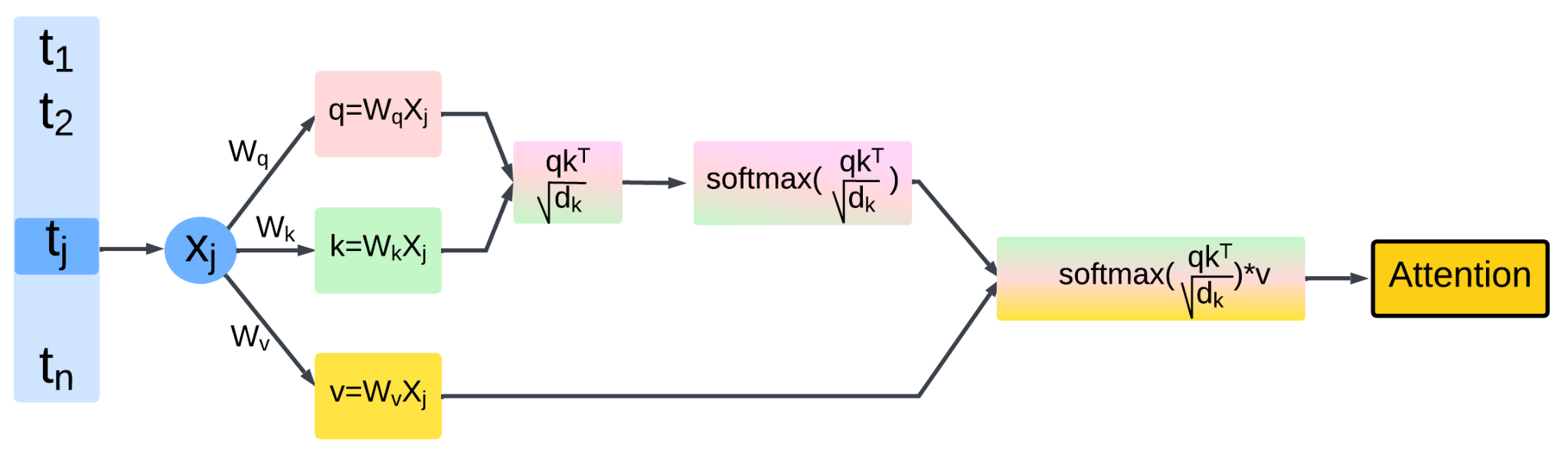


**Figure S6.** Conceptual representation of an attention (scaled dot product) mechanism. The blue bar represents a sequential input. The terms “W” indicates learnable parameters in the model.


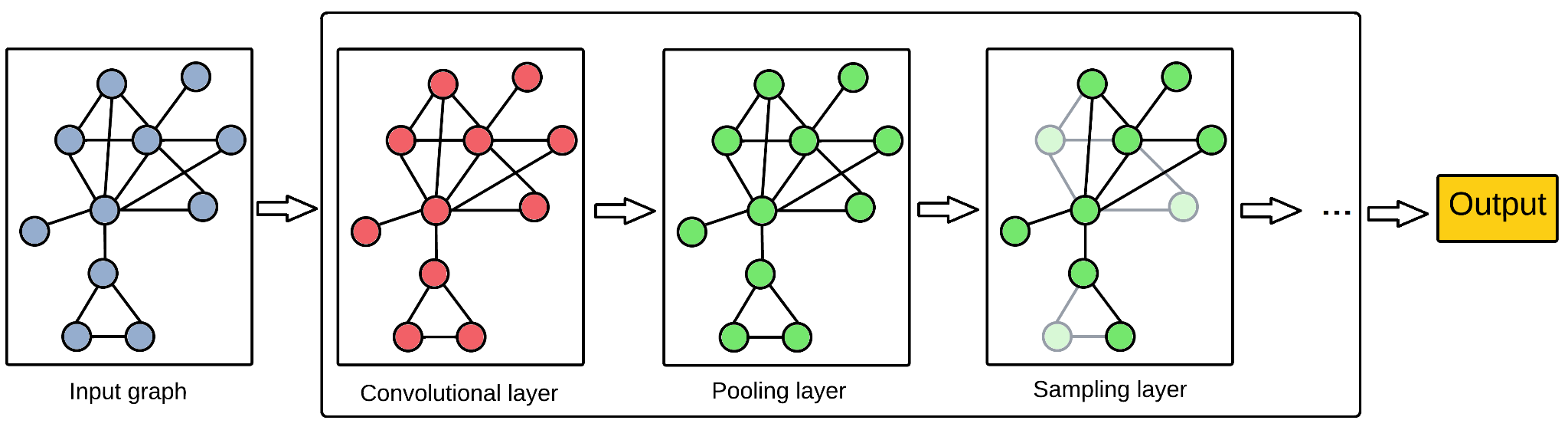


**Figure S7.** Conceptual representation of a graph neural network. The change of color from one layer to another indicates the extraction of features from the neighbors of the elements after convolution, or pooling operators. Sampling operator extracts a subgraph from the complete structure.


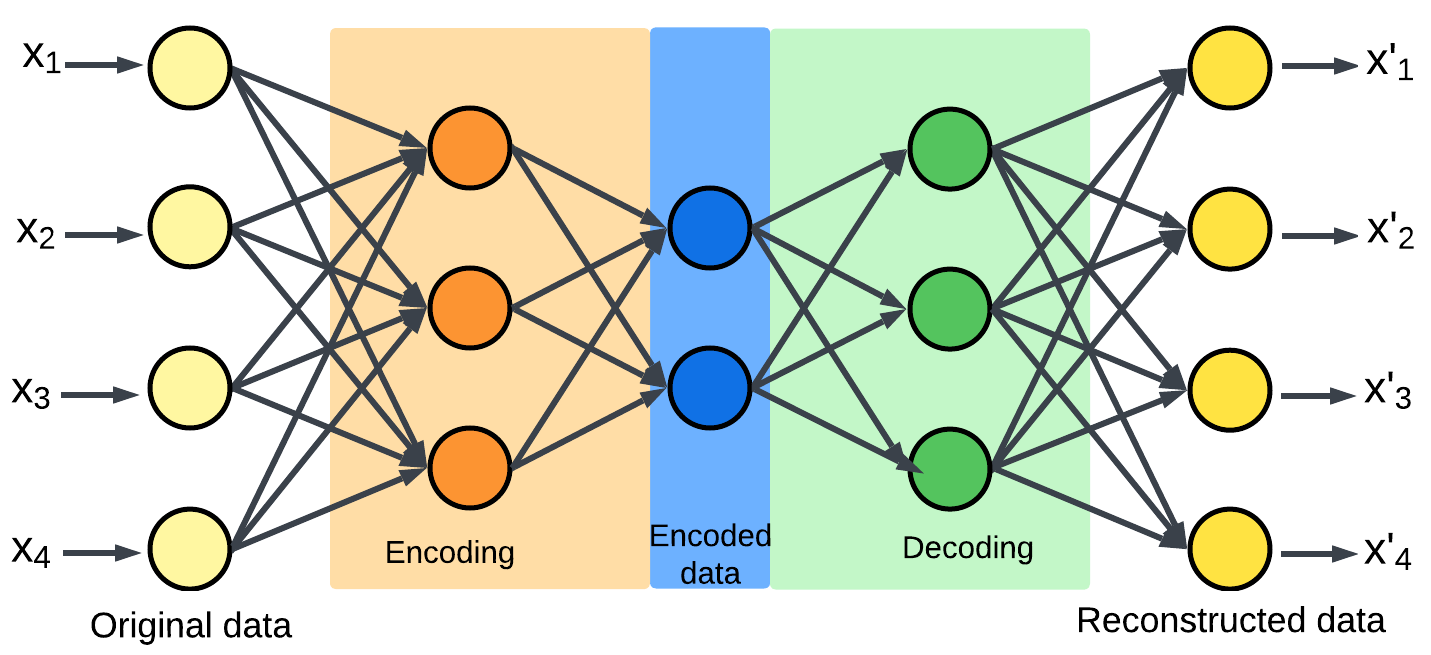


**Figure S8.** Typical architecture of an autoencoder. It consists of an autoencoding stage, a ‘bottleneck’ representing the encoded data, and a decoding stage where original data are reconstructed from the encoded data in their latent representation.

~~
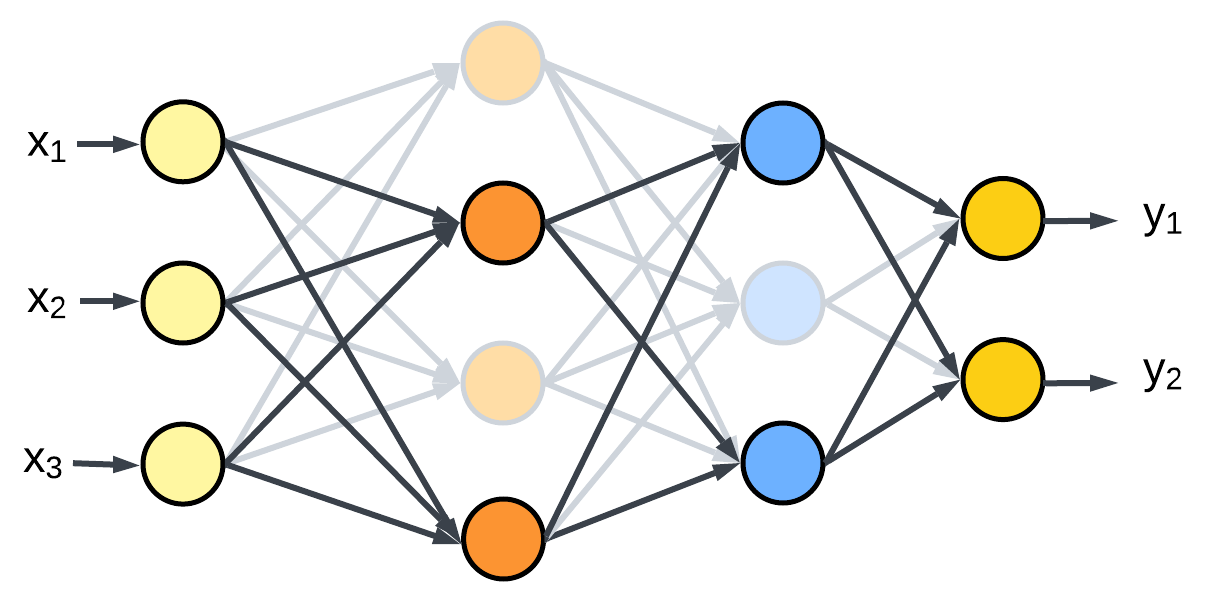
~~

**Figure S9.** Example of dropout applied to an MLP. The watermark wise units indicate they have been deactivated and no longer participate in the learning process during a specific iteration in the training of the model.

| **Table S1.** Performance of Stacked LSTM (Long-short term memory) and Temporal Attention compared to Lasso and SVR-RBF (taken from Shook et al. 2020). | | | | | | | |
| --- | --- | --- | --- | --- | --- | --- | --- |
| **T_x_** | **Model** | **Weather Variables** | | | **Including A II** | | |
|  |  | RMSE | MAE | R^2^ Score | RMSE | MAE | R^2^ Score |
| **7** | LASSO | 14.465±0.000 | 11.485±0.000 | 0.184±0.000 | 14.463±0.000 | 11.485±0.000 | 0.185±0.000 |
|  | SVR-RBF | 8.465±0.000 | 6.285±0.000 | 0.720±0.000 | 7.913±0.000 | 6.012±0.000 | 0.755±0.000 |
|  | Stacked LSTM | 8.290±0.021 | 6.191±0.007 | 0.731±0.001 | 7.257±0.025 | 5.470±0.024 | 0.794±0.001 |
|  | Temporal Attention | 8.292±0.018 | 6.172±0.027 | 0.731±0.001 | 7.243±0.037 | 5.453±0.030 | 0.795±0.001 |
| **15** | LASSO | 13.732±0.000 | 10.911±0.000 | 0.265±0.000 | 13.729±0.000 | 10.912±0.000 | 0.266±0.000 |
|  | SVR-RBF | 8.438±0.000 | 6.256±0.000 | 0.722±0.000 | 7.835±0.000 | 5.943±0.000 | 0.760±0.000 |
|  | Stacked LSTM | 8.284±0.029 | 6.176±0.034 | 0.732±0.002 | 7.248±0.020 | 5.460±0.015 | 0.795±0.001 |
|  | Temporal Attention | 8.284±0.017 | 6.182±0.035 | 0.732±0.001 | 7.226±0.017 | 5.441±0.013 | 0.796±0.001 |
| **30** | LASSO | 12.811±0.000 | 9.995±0.000 | 0.360±0.000 | 12.790±0.000 | 9.987±0.000 | 0.363±0.000 |
|  | SVR-RBF | 8.460±0.000 | 6.282±0.000 | 0.721±0.000 | 7.875±0.000 | 5.976±0.000 | 0.758±0.000 |
|  | Stacked LSTM | 8.283±0.005 | 6.178±0.004 | 0.732±0.000 | 7.276±0.050 | 5.484±0.049 | 0.792±0.002 |
|  | Temporal Attention | 8.303±0.019 | 6.200±0.013 | 0.730±0.001 | 7.239±0.032 | 5.441±0.028 | 0.795±0.002 |

| **Table S2. a)** Predictive ability in maize for random forest against tab-DNN in terms of root mean squared error (RMSE), relative RMSE (RMSE %), and R2 (coefficient of determination) for validation and holdout datasets. Bolded letters indicate the model with better performance; **b)** Comparison of performance of variations of the multimodal tab-DNN and sp-DNN fusion approach in terms of RMSE, RMSE% and R2. (taken from Danilevicz et al., 2021). | | | | | | | | |
| --- | --- | --- | --- | --- | --- | --- | --- | --- |
| **A** | **Model** | **Feature**  **Embedding** | **Validation dataset** | | | **Holdout dataset** | | |
|  |  |  | **RMSE** | **RMSE%** | **R^2^** | **RMSE** | **RMSE%** | **R^2^** |
|  | RF | One-hot  Embedding | **1.46±0.08** | **10.35±0.54** | **0.55±0.05** | **1.43** | **10.11** | **0.53** |
|  | tab-DNN | Embedding | 1.67±0.04 | 11.87±0.33 | 0.41±0.02 | 1.77 | 12.57 | 0.27 |
| **B** | **Multimodal Framework** | | **Validation dataset** | | | **Holdout dataset** | | |
|  |  |  | **RMSE** | **RMSE%** | **R^2^** | **RMSE** | **RMSE%** | **R^2^** |
|  | **Feature**  **Fusion** | Tabular module | 1.29±0.20 | 9.15±1.40 | 0.64±0.11 | 1.53 | 10.84 | 0.47 |
|  |  | Spectral module | 1.27±0.17 | 0.02±1.22 | 0.65±0.10 | 1.14 | 8.06 | 0.71 |
|  |  | Fusion module | 1.16±0.05 | 8.24±0.38 | 0.71±0.02 | 1.17 | 8.27 | 0.69 |
|  |  | Weighted prediction | **1.13±0.04** | **8.00±0.31** | **0.73±0.016** | **1.07** | **7.60** | **0.73** |
|  | **Feature Fusion**  **with**  **pre-trained**  **modules** | Tabular module | 1.29±0.21 | 9.10±1.50 | 0.64±0.12 | 2.16 | 15.31 | -0.07 |
|  |  | Spectral module | 1.27±0.18 | 8.96±1.31 | 0.65±0.11 | 1.19 | 8.44 | 0.67 |
|  |  | Fusion module | 1.14±0.04 | 8.09±0.25 | 0.72±0.02 | 1.22 | 8.63 | 0.66 |
|  |  | Weighted prediction | **1.12±0.02** | **7.90±0.16** | **0.74±0.01** | **1.21** | **8.55** | **0.66** |

| **Table S3.** Results from the comparison of six models by applying 10-fold cross validation. The metrics used were the coefficient of determination (R2), the mean average error (MAE), and the root mean squared error (RMSE). Models with the best performance are in bold letters. (taken from Måløy et al., 2021) | | | |
| --- | --- | --- | --- |
| **Model** | **R^2^** | **MAE** | **RMSE** |
| BGLR-RKHS (only SNPs) | 0.076 | 128.77 | 149.78 |
| CNN-MLP | 0.807 | 55.05 | 71.63 |
| ResNet-MLP | 0.690 | 72.55 | 90.79 |
| Plain Performer | 0.815 | 54.61 | 70.34 |
| Historical Performers | **0.820** | 53.61 | 69.29 |
| Multimodal Performer | **0.820** | **53.11** | **69.05** |

| **Table S4.** Accuracy, computed as the mean prediction accuracy from 20 replications. Comparison of NN-GBLUP (mixed effects based neural network) against single-step approach (Christensen et al.; 2021). The model was performed for several simulated proportions of omics missing data. (taken from Zhao et al.; 2022). | | | | | | | | | |
| --- | --- | --- | --- | --- | --- | --- | --- | --- | --- |
| **Missing omics data in training dataset (%)** | | | | | | | | | |
| **Method** | 0% | 10% | 30% | 50% | 70% | 80% | 90% | 95% | 99% |
| **Single-step**  **approach** | 0.577 | 0.562 | 0.537 | 0.523 | **0.495** | 0.474 | 0.448 | 0.409 | 0.355 |
| **NN-GBLUP** | **0.578** | **0.568** | **0.542** | **0.527** | 0.493 | **0.481** | **0.478** | **0.453** | **0.419** |

| **Table S5.** Performance on Test Set in Root Mean Squared Error (RMSE), Normalized Root Mean Square Error (nRMSE) and Pearson’s Correlation Coefficient (PCC) for all models (taken from Kick et al., 2023). | | | | |
| --- | --- | --- | --- | --- |
|  | **Model** | **RMSE** | **nRMSE** | **PCC** |
| **Genomic data** | Intercept | 1.088074 | 16.70137 |  |
|  | LM | 1.106886 | 16.99013 | 0.158382 |
|  | BLUP | 1.10248 ± 0.000124 | 16.92249 ± 0.001905 | 0.140023 ± 0.000315 |
|  | kNN | 1.078049 ± 2.10E-05 | 16.5475 ± 0.000323 | 0.153079 ± 9.82E-05 |
|  | RNR | 1.162622 ± 0.012629 | 17.84564 ± 0.193846 | 0.120362 ± 0.004536 |
|  | RF | 1.105258 ± 0 | 16.96514 ± 0 | 0.153836 ± 3.16E-17 |
|  | SVR | 1.219457 ± 0.048874 | 18.71803 ± 0.750191 | 0.059061 ± 0.082288 |
|  | DNN-CO | 1.100742 ± 0.009229 | 16.89582 ± 0.141659 | 0.149913 ± 0.016833 |
| **All modalities** | Intercept | 1.088074 | 16.70137 |  |
|  | LM | 0.972882 | 14.93323 | 0.389471 |
|  | BLUP | 0.937387 ± 0.057676 | 14.3884 ± 8.885301 | 0.461141 ± 0.091121 |
|  | kNN | 1.063364 ± 1.88E-06 | 16.32208 ± 2.89E-05 | 0.231872 ± 2.78E-06 |
|  | RNR | 1.09015 ± 1.40E-16 | 16.73324 ± 1.12E-15 | 0.176418 ± 3.01E-16 |
|  | RF | 1.106942±0.002913 | 16.99099 ± 0.044711 | 0.290892 ± 0.009171 |
|  | SVR | 1.040812 ± 0.041922 | 15.97593 ± 0.643488 | 0.29396 ± 0.066996 |
|  | DNN-CO | 0.948143 ± 0.01286 | 14.5535 ± 0.197387 | 0.426265 ± 0.020095 |
|  | DNN-SO | 1.023853 ± 0.034579 | 15.71561 ± 0.53077 | 0.272062 ± 0.084996 |

| **Table S6.** Results of 5FCV for thousand grain weight (TWG) for **A)** the GBLUP approach and **B)** for the MMDL approach for the dataset 1 (DS1) (taken from Montesinos-López et al., 2023). | | | |
| --- | --- | --- | --- |
| **MODEL** | **nRMSE (mean ± SD)** | **PCC (mean ± SD)** | **NDVIs form** |
| **A. GBLUP for TGW** |  |  |  |
| Year+G | 0.0663 ± 0.0089 | 0.8116 ± 0.0195 |  |
| Year+G+NDVI_GF | 0.0664 ± 0.0091 | 0.8112 ± 0.0209 | Averaged |
| Year+G+NDVI_GF | 0.0666 ± 0.0089 | 0.8095 ± 0.0198 | Aligned |
| Year+G+NDVI_VG | 0.0664 ± 0.009 | 0.8111 ± 0.0199 | Averaged |
| Year+G+NDVI_VG | 0.0671 ± 0.0087 | 0.8064 ± 0.0189 | Aligned |
| Year+G+NDVI_VG_GF | 0.0664 ± 0.009 | 0.8108 ± 0.0202 | Averaged |
| Year+G+NDVI_VG_GF | 0.0673 ± 0.0087 | 0.8047 ± 0.0196 | Aligned |
| Year+G+Year x G_i | 0.0665 ± 0.0095 | 0.81 ± 0.0227 |  |
| Year+G+Year x G_i+NDVI_GF | 0.0665 ± 0.0097 | 0.81 ± 0.0241 | Averaged |
| Year+G+Year x G_i+NDVI_GF | 0.0667 ± 0.0094 | 0.8086 ± 0.0223 | Aligned |
| Year+G+Year x G_i+NDVI_VG | 0.0665 ± 0.0095 | 0.81 ± 0.0229 | Averaged |
| Year+G+Year x G_i+NDVI_VG | 0.0672 ± 0.0094 | 0.8056 ± 0.0222 | Aligned |
| Year+G+Year x G_i+NDVI_VG_GF | 0.0665 ± 0.0097 | 0.8097 ± 0.0239 | Averaged |
| Year+G+Year x G_i+NDVI_VG_GF | 0.0674 ± 0.0094 | 0.8039 ± 0.0223 | Aligned |
| **B. MMDL for TGW** |  |  |  |
| Year+G | 0.0744 ± 0.0081 | 0.7608 ± 0.0158 |  |
| Year+G+NDVI_GF | 0.0722 ± 0.0078 | 0.7888 ± 0.0215 | Averaged |
| Year+G+NDVI_GF | 0.0802 ± 0.0076 | 0.7478 ± 0.0707 | Aligned |
| Year+G+NDVI_VG | 0.0741 ± 0.0067 | 0.7617 ± 0.0333 | Averaged |
| Year+G+NDVI_VG | 0.0708 ± 0.0076 | 0.7917 ± 0.0277 | Aligned |
| Year+G+NDVI_VG_GF | 0.0695 ± 0.0099 | 0.8054 ± 0.03 | Averaged |
| Year+G+NDVI_VG_GF | 0.0724 ± 0.007 | 0.7726 ± 0.0336 | Aligned |

**GRID Search and Bayesian Optimization methods**

Grid search is a method that treats hyperparameter space—the set of all possible values for hyperparameters—as a Cartesian product of individual sets of possible values for each hyperparameter. To implement grid search, numerical sets are converted into lists of values, often regularly spaced values in the logarithmic domain of the hyperparameters. Finding optimal hyperparameter values involves exhaustively searching through the Cartesian product (grid of values) formed by these lists.

However, grid search suffers from a significant drawback: it scales exponentially poorly as the number of hyperparameters increases (Bengio, 2012). An alternative approach is random search, which replaces the regular grid with a random sampling of points in the hyperparameter space. Experimental observations by Bergstra and Bengio (2012) suggest that random search can be significantly more efficient than grid search.

Bayesian optimization offers another method. It is a probabilistic optimization technique aimed at minimizing a complex, black-box objective function that lacks a closed-form solution. The hyperparameter space in Bayesian optimization can include continuous, integer, or categorical values. During optimization, Bayesian optimization constructs a surrogate function—a probabilistic model defining a distribution over the objective function for approximation. Additionally, an acquisition function is used to evaluate the effectiveness of evaluations at any point in the hyperparameter space (Masum et al., 2021). Python provides several modules designed for parameter tuning using these methods, such as hyperopt (Bergstra et al., 2013), hyperas (Pumperla, 2019), bayes_opt (Nogueira, 2020), and Optuna (Akiba et al., 2019).

**Details of implementation on MMDL applied to GS.**

The network proposed by Sharifi-Noghabi et al. (2019) consists of feedforward encoding sub-networks (encoders), one dedicated to each input omics data type. Each encoder incorporates fully connected layers with ReLU activation functions. Additionally, dropout is applied in each sub-model to regularize the overall network, while batch normalization (scaling and centering based on mean and standard deviation) is used to enhance the training process. The features learned from these encoding sub-networks are concatenated into a single representation. This representation undergoes further smoothing via an $l_{2}$ normalization layer. The resultant features are then input to a classifier responsible for predicting drug responses.

In MOLI, the final sub-network uses a sigmoid function in its classification layer and integrates dropout and weight decay for regularization. The entire network is trained using a combined cost function that includes both classification loss and triplet loss. Variants of MOLI are compared against a model proposed by Geeleher et al. (2017). This baseline model employs ridge regression solely on gene-expression data, non-negative matrix factorization (NMF) with early fusion, MLP, and unimodal MOLI.

The model proposed by Nguyen et al. (2021) comprises four primary layers: an input layer that integrates SNPs and genes, a transparent layer replicating gene nodes, a series of hidden layers, and a phenotype layer for output. Connections between the input and transparent layers are established using a gene regulatory network (GRN) and existing eQTLs knowledge, which reflects regulatory relationships among genes and from SNPs to genes. This approach, termed Biological Dropconnect, selectively removes links unsupported by eQTLs or GRNs instead of randomly deactivating such connections. This biological network structure enhances interpretability by mimicking natural biological mechanisms, allowing for the identification of significant features and pathways influencing prediction outcomes. Moreover, the model employs $l_{1}$ regularization (lasso) in the weights of the biological Dropconnect layer to emphasize more relevant features while reducing less relevant ones. Their fusion approach can be categorized as early fusion. Specifically, genomic (SNPs) and transcriptomic (gene expression) matrices, denoted as G and E, are transposed and concatenated to form $X=[G^{T}E^{T}]^{T}$, which serves as the input to the model.

The MMDL approach proposed by Venugopalan et al. (2021) employs stacked denoising autoencoders for Electronic Health Records (EHR) and Single Nucleotide Polymorphisms (SNPs), alongside a 3-dimensional Convolutional Neural Network (CNN) for MRI images. In cases where specific modality data (MRI, EHR, SNPs) was unavailable, patient data were represented as zeros in the deep learning (DL) models. Supervised Learning (SL) models such as k-Nearest Neighbors (k-NN), One-vs-One coding Support Vector Machines (SVM), Random Forests, and Decision Trees were used as final decision layers to make predictions. Performance metrics including accuracy, precision, recall, and F1 scores from internal cross-validation were used to compare model performance.

Despite Wang et al. (2021) using data types like transcriptomic, epigenomic, and non-coding RNA, which are not strictly genomic, we include this study because these data types can be integrated with genomic information within the same model. Each data type underwent processing in a modality graph using Graph Convolutional Networks (GCN), and the results from each modality were fused using a cross-omics discovery tensor. Subsequently, this tensor was processed by a View Correlation Discovery Network (VCDN), comprising a Multilayer Perceptron (MLP), to classify disease status. Performance evaluation for binary classification tasks included accuracy (ACC), F1 score (F1), and Area Under the Receiver Operating Characteristic Curve (AUC). For multi-class classification tasks, evaluation metrics included accuracy (ACC), average weighted F1 score (F1_weighted), and macro-averaged F1 score (F1_macro). Results were reported as the mean and standard deviation across five randomly generated training and testing splits. Additionally, multi-class classification performance was assessed using average weighted AUC score (AUC_weighted) and macro-averaged AUC score (AUC_macro).

In Shook et al. (2021), the dataset (after cleansing) comprised 103,365 performance records spanning 13 years for 5,839 distinct genotypes. Each record contained a 214-day multivariate time-series capturing a crop-growing season (from April 1 to October 31), with each day characterized by 7 weather variables. The dataset also included a maturity group factor. To enhance the model's applicability, genotype-specific criteria were employed, and mean location yield across genotypes was calculated. Due to limited molecular marker data availability, a comprehensive pedigree was constructed, resulting in a 5,839×5,839 correlation matrix. Genotypes were grouped into 20 clusters based on their developmental source, using the K-means algorithm for clustering. Each genotype was assigned to one of these 20 clusters, and the cluster ID was integrated into the yield prediction model. All models were trained with varying input sequence lengths (7, 15, and 30 days), considering only weather variables, alongside genotype cluster and maturity group information.

In Danilevicz et al. (2021), the tab-DNN model incorporates embedding layers for each of its five information channels, followed by dropout and batch normalization layers. The outputs then pass through two consecutive blocks featuring batch normalization, dropout, linear transformations, and ReLU activations. Finally, linear and sigmoid activations are applied for the final output. Notably, tab-DNN operates as a MMDL model in itself. On the other hand, sp-DNN is essentially a 2D convolutional network (CNN). The multimodal approach involves omitting the final decision layers from both tab-DNN and sp-DNN, concatenating the outputs from both models, and processing them through linear, ReLU, and linear activation functions to produce the final output. Four multimodal fusion approaches were evaluated with and without pre-trained modules: co-learning (simultaneous training) for tabular data and multispectral images, fusion layer, and weighted prediction. In both scenarios, with and without pre-trained modules, weighted prediction demonstrated the best performance, as illustrated in **Table 2**.

In Måløy et al. (2021), the evaluated models undergo assessment via 10-fold cross-validation using metrics such as the coefficient of determination (R2), mean average error (MAE), and root mean squared error (RMSE). The authors highlight that visualizing the self-attention maps of a Multimodal Performer network in this study demonstrates how the model establishes meaningful connections between genotype and weather data. These insights can empower breeders to make informed decisions in breeding processes, potentially reducing the length of breeding cycles.

The study by Wang and Chen (2022) diverges from others reviewed in this section by incorporating phenotypic data alongside genomic data. It was included due to its integration of genomic data with other modalities for predictive purposes. The subnetwork dedicated to genomic data combines CNN and LSTM networks to extract high-level feature representations from personal genome sequences in epigenetic regions. Conversely, the subnetwork for epigenetic data employs an MLP to generate feature representations for individual traits. A tensor fusion layer is subsequently used to capture both the individual modality representations and interactions between feature maps generated by these subnetworks. This layer integrates information from both modalities to produce a final feature map used for predictions. The study leveraged data from the ROSEMAP study (De Jager et al., 2018), including whole-genome sequencing (WGS) data, H3K9ac ChIPseq data, 450K DNA methylation array data derived from the dorsolateral prefrontal cortex, and phenotypic data from 202 AD patients.

Sharma et al. (2022) proposes a natural language processing (NLP)-based model aimed at capturing interactions between genomic (G), environmental (E), and management (M) data by treating DNA as natural language. Prior to modeling, feature selection is applied to SNPs data using LightGBM. A convolutional neural network (CNN) is employed to capture inter-genomic interactions. An inter-modalities attention module (Tan and Bansal, 2019) is utilized to capture GxE interactions, specifically considering weather conditions as contextual features for SNPs. Each of the six weather features (solar radiation, vapor pressure, precipitation, maximum and minimum temperature, and wind speed for each day during the planting season) is individually processed using CNNs. Soil data undergoes processing through a series of fully connected layers with ReLU activation functions, similar to the treatment of field management data.

The fusion sub-network consists of multiple ReLU activation and dropout layers. DeepG2P is benchmarked against a deterministic crop growth model (AutoCGM), GEBLUP, and the CNN model (CNN-21) proposed by Washburn et al. (2021) using Root Mean Squared Error (RMSE) and Pearson correlation coefficient (PCC). Model performance is evaluated through cross-validation by environment split, where test and validation pairs are excluded from training to assess generalization to unseen environments, and through hybrid split, where seeds are clustered and some clusters are used for testing and validation while the rest are for training.

In Kick et al. (2023), the proposed deep neural networks (DNNs) utilize 1-d convolutional layers to capture time-dependent features from weather data, while stacked fully connected layers handle other data modalities. The study employs two training strategies for GEM models: consecutive optimization (CO), where hyperparameters are tuned individually for yield prediction from each data source before optimizing for interaction layers, and simultaneous optimization (SO), which optimizes all hyperparameters concurrently, encompassing both individual modalities and inter-modality interactions.

Train/test splits were randomly generated while ensuring each location-year combination was exclusively in either the testing or training set. To ensure comprehensive evaluation, adjacent experimental sites were grouped to create training and testing sets, assessing the proposed models using time-series data represented as clusters. Evaluation metrics included Root Mean Squared Error (RMSE), normalized RMSE (NRMSE), and Pearson’s Correlation Coefficient (PCC) for comparison.

The multimodal approach proposed in Montesinos-López et al. (2023) was applied exclusively to Dataset 1 (DS1), which comprises data from 350 wheat cultivars and accessions from a wheat gene bank, evaluated by the Global Wheat Program at the International Maize and Wheat Improvement Center (CIMMYT) over two years (2015–2016 and 2016–2017). DS1 includes information on two traits, grain yield (YLD) and thousand grain weight (TGW), under favorable conditions (fully irrigated, timely sown). Genomic data and normalized difference vegetation index (NDVI) values served as covariates, measured multiple times during vegetative (VG) and grain filling (GF) periods. Measurements were aligned to the same date each year (aligned) or averaged across both periods.

The full linear model incorporated fixed effects for years, random effects of lines, random effects for the year × line interaction, and linear combinations of NDVI covariates (averaged or all date measurements) measured across different moments during VG and GF periods. Separate models were developed for each trait through 5-fold cross-validation (5FCV). DS1 was divided into five balanced parts, with 80% used for training and 20% for testing. This process was repeated for each partition, evaluating performance and reporting average metric values.

Additionally, the model assessed prediction performance over a full year using data from other years (leave-one-year-out, LOO), while excluding corresponding terms in the model for that year. For the Multimodal Deep Learning (MMDL) approach, DS1 was treated similarly to the Genomic Best Linear Unbiased Predictor (GBLUP) approach. In 5FCV, the model included a Multilayer Perceptron (MLP) for each modality, with outputs concatenated and processed by a fully connected layer with L2 regularization [FCL(L2)] to obtain final predictions. Batch normalization (BN) was applied to the output of each layer before activation. For LOO, only genomic and NDVI data were utilized.

The model architecture included residual blocks in the form of FCL(L2) + BN + ReLU - FCL(L2) + BN before activation. Evaluation metrics included normalized Root Mean Squared Error (nRMSE) and PCC between observed and predicted values. However, for GBLUP, accuracy was assessed using 5FCV, and while the proposed model outperformed BM and SVR, the GBLUP model in DS1 showed a slight advantage over the MMDL approach when incorporating genomic and various NDVI measurements.

In Liu & Mei (2023), a Sparse Principal Component Analysis (SPCA) method (Zou et al., 2006) is employed for feature selection to extract essential features. Subsequently, a patient similarity network evaluates the similarity of the three omics feature matrices individually, resulting in three n × n matrices. This approach aims to integrate heterogeneous data effectively while maintaining interpretability, resulting in smaller matrices. The challenge of high dimensionality in data is successfully addressed by transforming high-dimensional omics data (hundreds of thousands of dimensions) into a few thousand dimensions of sample similarity matrices.

The model utilizes a 7-layer deep neural network that incorporates the n × 3n fused similarity networks for training. This neural network includes three one-dimensional convolutional layers, each followed by a max-pooling layer. A batch normalization layer follows the last convolutional layer. Additionally, the first two fully connected layers use the ReLU activation function, while the third fully connected layer adopts softmax.

PheGeMIL, proposed by Togninalli et al. (2023), processes four data modalities: multispectral images, thermal images, digital elevation models (DEM), and SNPs. Convolutional residual networks with 18 layers (ResNet-18) handle the first three modalities, while SNP data is processed by a fully connected MLP. The model incorporates attention mechanisms to emphasize the importance of each input during prediction. Genotypic information, consisting of 38,361 SNP features, undergoes feature selection using Lasso.

The model utilizes an attention-based aggregation mechanism to combine embeddings from images and the genotype MLP across all dates. This approach notably enhances prediction accuracy, particularly when accounting for environmental effects. Despite integrating multiple channels, the most substantial improvement arises from incorporating genotypic information, suggesting that additional data channels do not necessarily significantly enhance predictive ability.

Chandrashekar et al. (2023) developed DeepGAMI, a deep learning model designed for brain disorder prediction that integrates multiple data modalities, emphasizing interpretability and managing incomplete data. The model leverages functional genomic information, including eQTLs and gene regulation, to guide connections within the model, enhancing interpretability. To handle missing modalities, DeepGAMI incorporates an Auxiliary Learning Layer (ALL) mechanism for cross-modal imputation.

The model adopts an early fusion approach where each modality undergoes preprocessing through a Biological Dropconnect Layer, which selectively deactivates synaptic connections based on biological insights. Extracted features from each modality are then used to train the ALL mechanism and are concatenated into a unified vector. This vector serves as input to a multi-layer perceptron (MLP) that predicts the phenotype.

In scenarios with incomplete data, the ALL mechanism imputes missing modalities, ensuring that the combined feature vector remains comprehensive for feeding into the MLP. This framework not only enhances interpretability by leveraging biological knowledge but also addresses the challenge of incomplete data, thereby improving the robustness of disease prediction.

**A simple MMDL example with Python code**

The Python code for implementing and evaluating the model's prediction performance for the heading trait using a fivefold cross-validation strategy is available through the following link . <https://github.com/osval78/Multimodal_Genetics_Example>

To facilitate the use of this code, comments were added to various important parts of the program. In general, the objective is that anyone can access the code and run a multimodal model with the different modalities they need to implement using their own data. First, install Python 3.10 with TensorFlow 2.10, and then install the required packages: pip install numpy pandas scikit-learn scipy matplotlib statsmodels patsy pyreadr bayesian-optimization.Then, modify the most important parts of the code that must be changed to at least run the model with your own data:

1. Line 81 of the code: Specify the suffix for the dataset names to be evaluated, which will be read on line 87. The name to be read results from combining ‘dat-Trait-’ with the suffix specified in the variable Traits = [] on line 81.
2. The ‘Pheno’ object, as defined in the comments on lines 87-89, needs to have column names that specify the following: GID, the environment (Env), and the values of the measured trait or response. The trait values are extracted on line 99, and the name must be one of those specified in Traits (line 81).
3. This example will incorporate two modalities in the predictor: the environment effects and the line effects through the genomic relationship matrix. For the environment effects, we can include the corresponding features by creating the design matrix of environments, specifying the column for the environment (Env) in the phenotypic data saved in dat_F (line 106). For the line effects, the comments on lines 87-89 need to be addressed. Additionally, the Pheno dataframe must contain the variable GID with the names of individual lines, as the code on line 116 requires this information to obtain the design matrix for the lines.
4. If more than two modalities are to be used in the model, list all of them separated by commas on line 159.
5. Furthermore, if the ‘Fold’ object defined in the comments on lines 87-89 is missing in the loaded .RData file, comment out lines 122-124 of the code example and uncomment lines 125-130 to use a 10-fold cross-validation partition specified on line 125 (change 10 to another value if needed).
6. The name of the folder where the outputs will be saved can be modified by changing ‘Outs’ on line 133 to the desired name. After the ‘best’ model is found using the Bayesian optimization algorithm, the predictions and the observed values of responses are saved in a .csv file with a name formatted as ‘Preds-Traits-?-??-PT-???’, where ? will contain the name of the corresponding Traits (see line 81), ?? will denote the model name (line 136) indicating the modalities involved (in this case, Env and GID), and ??? will indicate the fold corresponding to the prediction evaluations. This naming format is defined on line 240.

Finally, for each trait, the output folder will also include a summary of some evaluated metrics (Pearson’s correlation (Cor), mean squared error (MSE), and normalized mean squared error (NRMSE)) computed for each testing set (Fold), along with the partition indicator (PT) and other hyperparameter values specified by default or personally chosen by the user.

## 
